# Platinum–copper single atom alloy catalysts with high performance towards glycerol hydrogenolysis

**DOI:** 10.1038/s41467-019-13685-2

**Published:** 2019-12-20

**Authors:** Xi Zhang, Guoqing Cui, Haisong Feng, Lifang Chen, Hui Wang, Bin Wang, Xin Zhang, Lirong Zheng, Song Hong, Min Wei

**Affiliations:** 1 State Key Laboratory of Chemical Resource Engineering, Beijing Advanced Innovation Center for Soft Matter Science and Engineering, Beijing University of Chemical Technology, 100029 Beijing, P. R. China; 2 Beijing Research Institute of Chemical Industry, Sinopec Group, 100013 Beijing, P. R. China; 3 Institute of High Energy Physics, Chinese Academy of Sciences, 100049 Beijing, P. R. China

**Keywords:** Catalyst synthesis, Heterogeneous catalysis, Bioalcohols

## Abstract

Selective hydrogenolysis of biomass-derived glycerol to propanediol is an important reaction to produce high value-added chemicals but remains a big challenge. Herein we report a PtCu single atom alloy (SAA) catalyst with single Pt atom dispersed on Cu nanoclusters, which exhibits dramatically boosted catalytic performance (yield: 98.8%) towards glycerol hydrogenolysis to 1,2-propanediol. Remarkably, the turnover frequency reaches up to 2.6 × 10^3^ mol_glycerol_·mol_PtCu–SAA_^−1^·h^−1^, which is to our knowledge the largest value among reported heterogeneous metal catalysts. Both in situ experimental studies and theoretical calculations verify interface sites of PtCu–SAA serve as intrinsic active sites, in which the single Pt atom facilitates the breakage of central C–H bond whilst the terminal C–O bond undergoes dissociation adsorption on adjacent Cu atom. This interfacial synergistic catalysis based on PtCu–SAA changes the reaction pathway with a decreased activation energy, which can be extended to other noble metal alloy systems.

## Abstract (web summary)

Selective hydrogenolysis of biomass glycerol to propanediol is a promising route for the production of high-value chemicals but remains a challenge. Here, the authors find a PtCu single atom alloy catalyst exhibits remarkably boosted performance with a turnover frequency value of 2.6 × 10^3^ mol_glycerol_·mol_PtCu–SAA_^−1^·h^−1^.

## Introduction

With the huge consumption of limited reserves fossil fuels and increasing environmental issues, biomass, as the abundant carbon-neutral renewable resources, has attracted considerable attention in both fundamental study and industrial application. In this context, converting biomass-derived feedstocks (e.g., polyols, sugars) to clean fuels and fine chemicals through green catalytic processes has become a promising strategy^1^. In particular, glycerol is usually regarded as an important biomass-derived “platform” chemical, since it is the smallest and most representative polyol with a highly functionalized molecule^2,3^. Among various transformations approaches (e.g., hydrogenolysis, oxidation, and dehydration etc.), one of the most favorable processes is the highly selective of hydrogenolysis glycerol to 1,2-propanediol (1,2-PDO), which serves as a significant commodity chemical widely applied in the production of unsaturated polyester resin, deicing agent and cosmetics^4,5^. Normally, hydrogenolysis reaction undergoes splitting of C–O and/or C–C bonds and simultaneous addition of hydrogen to form a desired product^6,7^. Especially, selective breakage of one terminal C–O bond with the preservation of other C–O and C–C bond is highly crucial to produce 1,2-PDO, due to the coexistence of multiple hydroxyl groups and C–C bonds^8–10^. Along this line, considerable effort has been focused on exploration of heterogeneous catalysts towards this hydrogenolysis reaction, including non-noble metals (e.g., Co, Ni, and Cu), noble metals (e.g., Pd, Ru, and Pt), and bimetallic (e.g., RuFe, CuNi, and CuPd) catalysts^10–14^. Although much progress has been made, structure design and development of highly efficient catalysts to acquire high activity, selectivity and stability simultaneously, still remain a big challenge.

Generally, bimetallic alloy catalysts have shown great advantages in boosting catalytic performance in many reactions, compared with monometallic catalysts, due to their synergistic effect^15–17^. To maximize the utilization of predominant metal atom (especially noble metal), one of the extreme cases is to atomically disperse one active metal on the surface of the second one (e.g., single atom alloy, SAA)^18–21^. SAA catalysts has been proven to possess peculiar electronic and geometric features rather different from their constituent metals, which provide unique active centers and consequently change the reaction pathway^22–24^. So far SAA exhibits remarkable catalytic behavior in many chemical reactions (e.g., hydrogenation, hydrogenolysis)^25–30^. Currently, selective glycerol hydrogenolysis to 1,2-PDO is mainly performed over Cu-based catalysts, because of their strong tendency in cleaving the C–O bond and low scission ability for C–C bond, whereas suffers from an unsatisfactory activity and poor thermal stability^31,32^. In contrast, noble Pt-based catalysts exhibit superior activity but are prone to break C–C bond, resulting in a loss of selectivity^33,34^. Inspired by the above facts, if a unique bimetallic SAA catalyst is designed by distributing Pt atoms onto Cu nanoparticles to marry their advantages, both activity and selectivity would be guaranteed simultaneously.

Herein, we design and synthesize a PtCu single atom alloy (PtCu–SAA) catalyst based on structural transformation from hydrotalcite precursor (CuMgAl–LDH) followed by a galvanic replacement reaction to introduce Pt single atom onto the surface of Cu nanoclusters. A series of elaborate characterizations including AC–HAADF–STEM, in situ CO–DRIFTS and in situ EXAFS confirm the formation of SAA, where a few Pt atoms are absolutely isolated by Cu atoms. The PtCu–SAA exhibits extraordinary catalytic performance towards glycerol hydrogenolysis to 1,2-propanediol (conversion: 99.6%; selectivity: 99.2%), under mild reaction conditions (200 °C, 2 MPa). Most notably, the turnover frequency (TOF) value of PtCu–SAA reaches up to 2.6 × 10^3^ mol_glycerol_∙mol_PtCu–SAA_^−1^·h^−1^, which is 8–120 fold larger than that of metal catalysts ever reported. This is to our knowledge the highest reactivity among heterogeneous catalysts under identical reaction conditions. Furthermore, both in situ experimental studies and theoretical calculations substantiate that the Pt–Cu interface sites serve as intrinsic active centers: the single Pt atom facilitates the activation adsorption of central C–H bond in glycerol molecule, while the terminal C–O bond undergoes dissociation adsorption on adjacent Cu atoms. Consequently, this interfacial synergistic catalysis changes the reaction pathway with a lower activation energy, compared with traditional monometallic catalysts. Therefore, this work illustrates a successful paradigm for the design of SAA catalyst towards boosting polyols hydrogenolysis reaction, which shows potential application in biomass feedstocks to clean fuels and fine chemicals.

## Results

### Structural characterizations

**Fig. 1:**
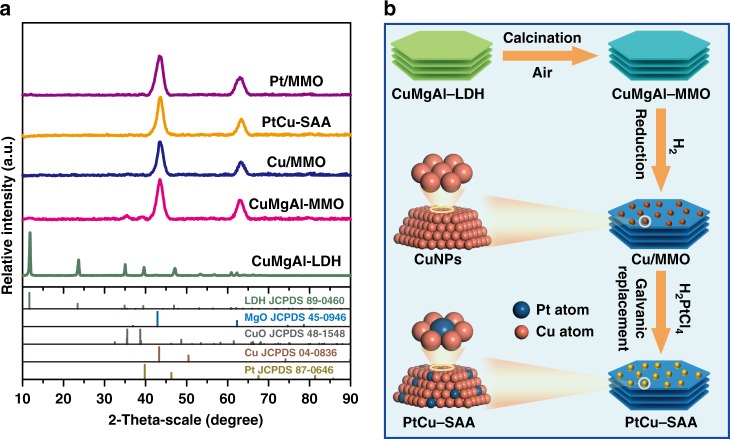
Synthesis and characterization of PtCu–SAA from LDHs precursor. **a** In situ XRD patterns of various samples; **b** a schematic illustration for the preparation of PtCu–SAA.

The XRD patterns of CuMgAl–LDH precursors in Fig. 1a show series of characteristic reflections at 2*θ* 11.9°, 23.5°, and 34.9°, indexed to the (003), (006), and (012) of a typical carbonate-containing LDH phase, respectively^35^. The subsequent calcination treatment (air, 500 °C) results in a structural topotactic transformation from hydrotalcite to mixed metal oxides (CuMgAl–MMO). The XRD patterns display two weak peaks (35.5°, 38.7°) attributed to the (111) reflection of CuO (JCPDS 048–1548), and other two strong peaks (42.9°, 62.3°) indexed to high-crystallinity MgO (JCPDS 045–0946). No reflection of Al_2_O_3_ was observed, implying an amorphous phase^36^. Afterwards, samples of CuMgAl–MMO were carefully reduced in a H_2_/Ar atmosphere to obtain Cu nanoclusters supported on oxides matrix (Cu/MMO). Finally, PtCu–SAA sample was obtained by a galvanic replacement reaction to introduce platinum single atom onto the surface of Cu NPs (Fig. 1b). Meanwhile, the Pt–Cu alloy samples with various atomic ratios were synthesized as control samples (PtCu–NPs). It is noted that Cu/MMO and PtCu–SAA show rather identical XRD patterns without Cu or CuO phase, implying a high dispersion degree of copper species. Additionally, PtCu–SAA and PtCu–NP–1 sample (Supplementary Fig. 1a) do not display Pt reflection while the PtCu–NP–2 sample shows three weak peaks at 2*θ* 39.8°, 46.3°, and 81.3° indexed to the (111), (200), and (311) reflection of Pt (JCPDS 087–0646), due to the increase of Pt loading.

**Fig. 2:**
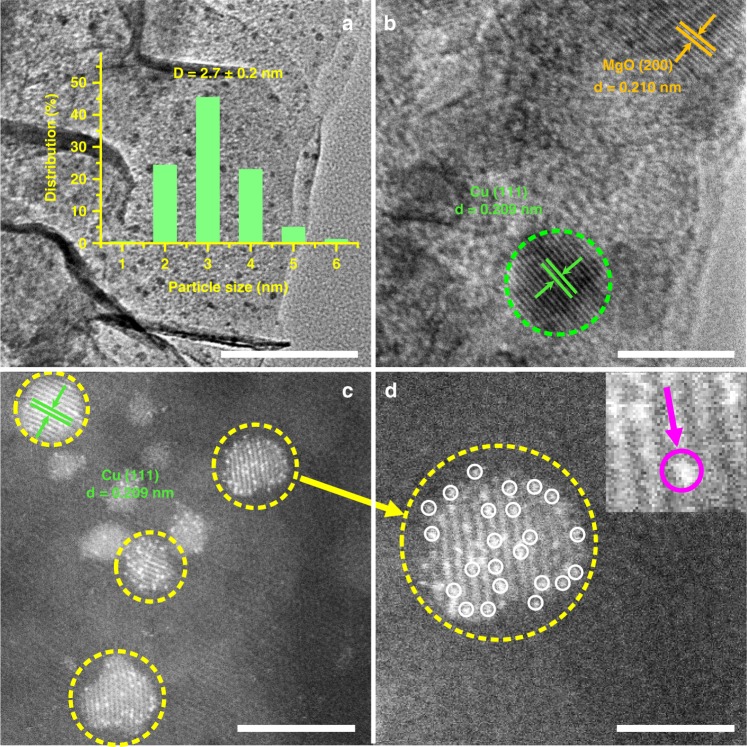
Identification of PtCu single atom alloy. **a** TEM image, **b** HRTEM image, **c** AC–HAADF–STEM image of PtCu–SAA, and **d** corresponding enlarged images. Scale bars: **a** 100 nm; **b** 5 nm; **c**, and **d** 2 nm.

HRTEM measurements were carried out to gain more spatially-resolved structure information on the Cu/MMO and PtCu–SAA samples. From the HRTEM images of both samples (Fig. 2a, b and Supplementary Fig. 2a, c), Cu NPs are well dispersed and embedded onto the MMO support with a similar content (∼3.2 wt.%) and a close particle size (∼2.7 nm). Moreover, two clear crystalline phases are identified for these two samples: Cu (111) plane (lattice spacing: 0.209 nm) and MgO (200) plane (lattice fringe: 0.210 nm), respectively. To unambiguously observe the Pt species, the aberration-correction high-angle annular dark-field scanning transmission electron microscopy (AC–HAADF–STEM) imaging technique was employed to distinguish Pt atom based on the difference in Z-contrast. The image of Pt/MMO samples displays both Pt nanoclusters and Pt single atoms (Supplementary Fig. 2b, d). In contrast, a number of bright and atom-sized features (Fig. 2c) attributed to individual Pt atoms can be discerned on the crystal surface of Cu in PtCu–SAA samples. Interestingly, these isolated Pt atoms are surrounded by Cu atoms in several different regions of the samples, rather than Pt nanoclusters (Supplementary Fig. 3). A typical AC–HAADF–STEM image of an individual Cu nanoclusters in Fig. 2d was displayed to highlight the surface Pt atoms. The randomly enlarged image further confirms the substitution of surface Cu atoms by isolated Pt atom, where the single Pt atom was clearly observed (marked by magenta circle and arrow). The lattice spacing of PtCu–SAA in Fig. 2c is 0.209 nm, in good agreement with that of pure Cu (111) in Cu/MMO sample. In contrast, three crystalline phases of PtCu–NPs sample are identified for PtCu alloy (Supplementary Fig. 1b–c): Cu (111) plane (expanded lattice spacing: 0.211 nm), Pt (111) and (200) plane (shrunk lattice fringe: 0.220 nm and 0.190 nm), respectively^37^. In addition, the energy dispersive spectroscopy (EDS) mapping images of PtCu–SAA samples (Supplementary Fig. 4) display Pt is mainly distributed on the surface of Cu nanoclusters and not supported on MgAl–MMO substrate. Moreover, the Cu dispersion (D_Cu_) of PtCu–SAA sample determined by classic N_2_O chemisorption, is significantly up to 50.7% (Table 1), similar to that of Cu/MMO (52.3%). The Cu/MMO and PtCu–SAA samples display similar reduction degree of Cu (~90%) within the range of reduction temperature (Table 1), indicating predominant Cu^0^ species accompanied with a little Cu^+^ species.

**Table 1:** Physicochemical parameters of samples.

Samples	BET	Cu content^a^	Pt content^a^	Mean size^b^	D_M_^c^	R_Cu_^d^
	(m^2^∙g^−1^)	(wt.%)	(wt.%)	(nm)	(%)	(%)
PtCu–SAA	168.4	3.2	0.21	2.7 (Cu)	50.7 (Cu)	88.2
Cu/MMO	153.2	3.3	−	2.9 (Cu)	52.3 (Cu)	90.8
Pt/MMO	123.1	−	0.29	1.4 (Pt)	80.1 (Pt)	−

^a^Contents of metal Cu and Pt were determined by ICP–AES

^b^Mean metal particle size of Cu and Pt were determined by TEM

^c^Dispersion of metallic Cu and Pt were measured based on N_2_O chemisorption and hydrogen oxygen (H_2_−O_2_) titration experiments, respectively

^d^Reduction degree of Cu was determined by H_2_–TPR measurements

**Fig. 3:**
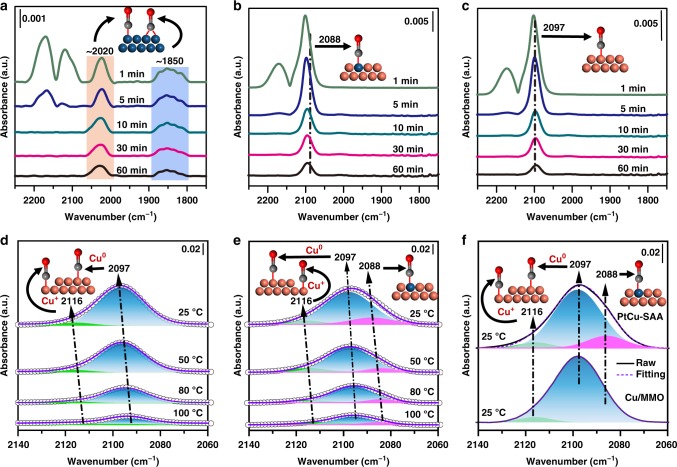
In situ infrared spectroscopy studies on PtCu–SAA surface. In situ CO-DRIFTS spectra of **a** Pt/MMO, **b** PtCu–SAA, and **c** Cu/MMO purging with helium as a function of time; the enlarged and Gaussian fitting spectra with a fixed peak position and FWHM of **d** Cu/MMO and **e** PtCu–SAA as a function of temperature (the black hollow circle: experimental data; the violet solid line: fitting curve); **f** in situ CO-DRIFTS spectra of Cu/MMO and PtCu–SAA at 25 °C (the black solid line: experimental data; the violet dotted line: fitting curve). Cu, orange, Pt, blue, C, gray, O, red.

In situ CO–DRIFTS measurements were performed to probe the atomic geometry configuration of Pt ensembles on the surface of Cu NPs, in comparison with Cu/MMO, PtCu–NPs, and Pt/MMO samples. As shown in Fig. 3a and Supplementary Fig. 1d–g, CO adsorption on Pt/MMO and PtCu–NPs sample produces a strong and broad vibration band at 2010–2060 cm^−1^ and another weak band at 1810–1880 cm^−1^, which are ascribed to linearly bonded CO at Pt^0^ sites and bridged-adsorbed CO at two adjacent Pt atoms, respectively^38^. The appearance of bridge-bonded CO signifies the existence of dimer or Pt clusters, in accordance with the AC–HAADF-STEM results (Supplementary Fig. 1b–c and 2d). However, the relative intensity of linear and bridged band (Supplementary Fig. 1d–g) is much weaker in PtCu–NP–1 than that in PtCu–NP–2, due to the Pt content. The results above combining with AC–HAADF–STEM and EDS mapping images (Supplementary Figs. 1, 5 and 6) indicate PtCu–NP–1 sample consists of principal PtCu single atom alloy, some PtCu alloy and Pt/Cu nanoclusters; while PtCu–NP–2 sample contains predominant PtCu alloy, some Pt/Cu nanoclusters and a few PtCu single atom alloy. In contrast, neither bridged- nor linearly bonded CO peaks are found over PtCu–SAA (Fig. 3b). Moreover, only one asymmetric absorption band between 2060 and 2140 cm^−1^ is observed, indicating the overlapping signals for CO adsorption between the isolated Pt atoms and Cu NPs (Fig. 3b–c). To clearly distinguish the overlapping peaks, in situ CO–DRIFTS spectrum dependent on temperature were recorded on PtCu–SAA, with Cu/MMO as a reference sample, which were fitted and deconvoluted via multi–peaks Gaussian fitting. The whole profiles of Cu/MMO are deconvoluted to a main peak at 2097 cm^−1^ and another weak peak at 2116 cm^−1^ (Fig. 3d), which are usually attributed to CO chemisorbed on Cu^0^ and Cu^+^ species, respectively^39–41^. Interestingly, an obvious shoulder peak (Fig. 3e, f) is observed for PtCu–SAA within 2080–2090 cm^−1^, compared with Cu/MMO samples at the same temperature. The whole profiles of PtCu–SAA were reasonably fitted and deconvoluted to three peaks as follows: an obvious shoulder peak centered at 2088 cm^−1^ and the two peaks analogous to Cu/MMO sample with same peak position and FWHM. This unique absorption band centered at 2088 cm^−1^ is assigned to the linear adsorption of CO on isolated single Pt atoms^29,42,43^. Hence, the results of in situ CO–DRIFTS confirm that Pt species exists as isolated atom in PtCu–SAA samples, in agreement with the above AC–HAADF–STEM observations.

**Fig. 4:**
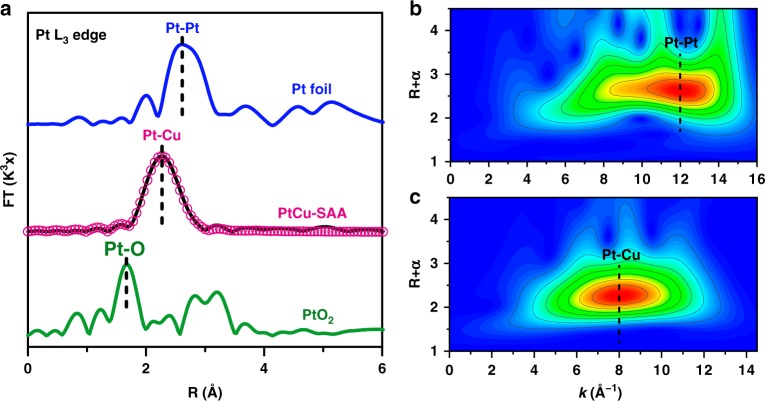
Characterization and identification of PtCu single atom alloy. **a** In situ Pt L_3_–edge EXAFS Fourier–transform spectra of Pt foil, PtCu−SAA (the empty magenta circle: fitting curve; the black solid line: experimental data) and PtO_2_ sample, the in situ EXAFS wavelet transforms spectra of **b** Pt foil and **c** PtCu–SAA, respectively.

As discussed above, infrared spectroscopy and electron microscopy provide useful information about surface and structure properties, but no Cu-containing and Pt-containing crystal phases were detected in the XRD patterns of PtCu–SAA sample (Fig. 1a), primarily due to the insensitivity of X-ray diffraction to low content and/or small clusters. Herein, we used in situ extended X-ray absorption fine spectroscopy (EXAFS) at the Pt-L3 edge and Cu-K edge to further confirm the atomically dispersed Pt and to determine the coordination structure of Pt and Cu. The Fourier transforms spectrum in the R space from the *k*^3^-weighted EXAFS of PtCu–SAA sample exhibits one prominent peak in the region ∼2.2–2.3 Å (Fig. 4a), which is located between PtO_2_ standard sample and Pt foil, indicating the formation of PtCu alloy^43^. Supplementary Table 1 lists the fitting results including coordination numbers (CN) and structural parameters. The Pt foil shows 12 Pt–Pt coordination at 2.77 Å; PtO_2_ displays 6 Pt–O coordination (2.05 Å) and 6 Pt–Pt coordination (3.09 Å). Surprisingly, regarding PtCu–SAA sample, only ~7.4 Pt–Cu coordination at 2.57 Å is identified; neither Pt–Pt nor Pt–O coordination contribution is detected. This demonstrates that predominant Pt are distributed as isolated and individual atom surrounded by Cu atoms rather than MgAl–MMO support.^22,23^ To further strengthen this result, the wavelet transforms (WT) analysis of Pt EXAFS oscillations was conducted, giving powerful resolution in both *k* and R spaces. As illustrated by the WT contour plots of Pt foil (Fig. 4b) and PtO_2_ (Supplementary Fig. 7), the intensity maxima at ~12 Å^−1^ and ~6 Å^−1^ are attributed to the Pt–Pt and Pt–O contributions, respectively. In contrast, for the WT contour plot of PtCu–SAA (Fig. 4c), one intensity maximum at near 8 Å^−1^ is exclusively observed, which is assigned to the Pt–Cu contribution^44^. Therefore, the results of AC–HAADF–STEM, in situ CO–DRIFTS and in situ XAFS characterizations verify the formation of PtCu single atom alloy with atomically dispersed Pt atoms on the surface of Cu nanoclusters while Pt deposited on MgAl–MMO support is not detected on PtCu–SAA samples.

### Catalytic performance for glycerol hydrogenolysis

The catalytic properties of monometallic samples (Cu/MMO and Pt/MMO) and various PtCu alloy samples (PtCu–SAA, PtCu–NPs) were investigated towards glycerol hydrogenolysis to 1,2-PDO. These three PtCu–SAA samples with different Pt/Cu ratios were synthesized to study the necessity of Pt single atom site (Supplementary Fig. 8). The initial activity shows an almost linear enhancement with the increase of Pt content from 0.10 wt.% (PtCu–SAA–1) to 0.15 wt.% (PtCu–SAA–2) and then to 0.21 wt.% (PtCu–SAA), and their selectivity maintains above 96%. This demonstrates the single atom alloy serves as intrinsic active site. Thus, the PtCu–SAA sample was chosen as the target catalyst due to its optimal catalytic performance.

**Fig. 5:**
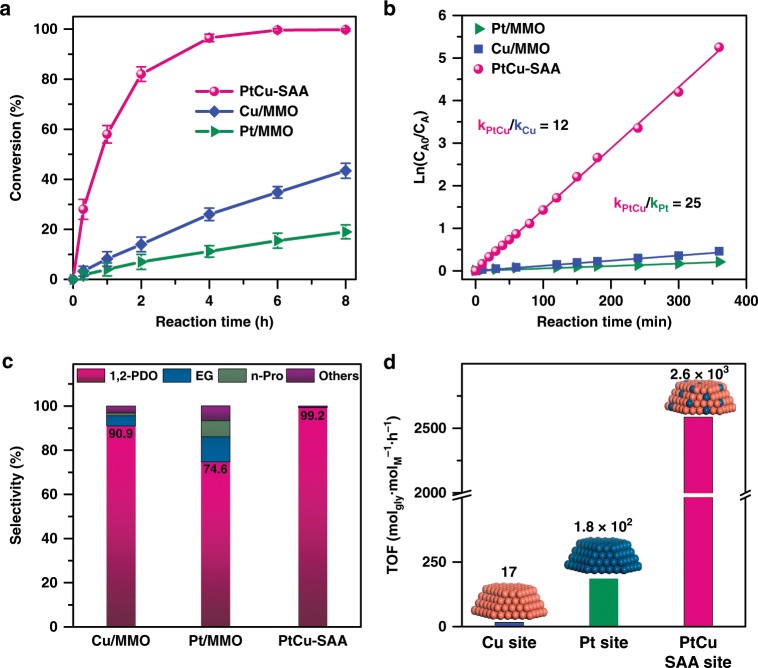
Reaction studies on glycerol hydrogenolysis to 1,2-PDO. Catalytic evaluation of PtCu-SAA and monometallic catalysts (Pt/MMO and Cu/MMO) toward glycerol hydrogenolysis to 1,2-PDO: **a** conversion versus reaction time, **b** ln (*C*_A0_/*C*_A_) versus reaction time, **c** the product selectivity, and **d** the TOF value determined as moles of initial glycerol converted per mole of exposed active sites per hour in the catalytic dynamic range. Reaction conditions: 10 mL of glycerol alcoholic solution (10 wt. %), 0.14 g of catalyst, 200 °C, and 2.0 MPa of H_2_ pressure. Error bars were defined as standard deviation, *n* = 3.

As shown in Fig. 5, both Pt/MMO and Cu/MMO catalysts give a low conversion (14.5% and 38.9%), and the 1,2-PDO selectivity reaches at a normal level (74.6% and 90.9%). The main by-products are ethylene glycol and *n*-propanol, resulting from the cleavage of C–C bond and excessive hydrogenolysis, respectively. Interestingly, PtCu–SAA sample displays significantly enhanced catalytic performance (conversion: 99.6%; selectivity: 99.2%). The high activity and selectivity, is superior to the previously reported bimetallic catalysts (Supplementary Table 3). Even at a low temperature (~120 °C), the PtCu–SAA catalyst also exhibits a satisfied catalytic performance (Supplementary Table 4). However, both the conversion and selectivity (Supplementary Table 2) show a sharp decrease from 99.6% and 99.2% (PtCu–SAA) to 89.5% and 90.4% (PtCu–NP–1), and then to 58.8% and 71.2% (PtCu–NP–2), respectively. This demonstrates PtCu single atom alloy as active sites affords higher catalytic performance than PtCu alloy and Pt/Cu nanoclusters. Furthermore, the plots of ln (*C*_A0_/*C*_A_) as a function of reaction time are straight lines starting with the origin (Fig. 5b), indicating a pseudo first-order reaction with respect to glycerol. The calculated rate constant for PtCu–SAA is 1.4 × 10^−2^ min^−1^, which is greatly larger than that for Cu/MMO (1.2 × 10^−3^ min^−1^) and Pt/MMO (5.7 × 10^−4^ min^−1^). The results show a significantly promoted catalytic performance with the synergistic effect between Cu and Pt.

To further reveal the unique intrinsic catalytic activity, the turnover frequency (TOF) was measured for glycerol hydrogenolysis to 1,2-PDO (Fig. 5d and Supplementary Table 3). The TOF value of Cu/MMO catalyst (17 mol_glycerol_∙mol_Cu_^−1^·h^−1^) is close to that of traditional Cu-based catalysts. The Pt/MMO catalyst shows a good TOF value of 1.8 × 10^2^ mol_glycerol_∙mol_Pt_^−1^·h^−1^ compared with other noble metal catalysts. Notably, the TOF value of PtCu–SAA catalyst dramatically reaches up to 2.6 × 10^3^ mol_glycerol_∙mol_PtCu–SAA_^−1^·h^−1^, calculated on the basis of moles of whole single atom alloy sites (with two atoms) on the surface of PtCu–SAA, which is ~150 and 14 times larger than monometallic Cu/MMO and Pt/MMO catalyst, respectively. It is worth mentioning that the TOF value of PtCu–SAA, to the best of our knowledge, is the highest level among all reported metal catalysts (Supplementary Table 3). Moreover, the 1,2-PDO productivity over PtCu–SAA reaches up to 384 _g1,2-PDO ∙gM_^−1^·h^−1^ (6 h, yield_1,2-PDO_: 98.8%) based on the mass of noble metal Pt, 58.6 _g1,2-PDO ∙gM_^−1^·h^−1^ (2 h, yield_1,2-PDO_: 81.7%) and 23.7 _g1,2-PDO ∙gM_^−1^·h^−1^ (6 h, yield_1,2-PDO_: 98.8%) based overall active metal (Pt and Cu) mass, which are much larger than that of reported metal catalysts under the similar conditions (Supplementary Table 3). In addition, the reusability of PtCu–SAA sample was tested by five successive hydrogenolysis recycling, giving a slight performance decrease within 4%. A series of elaborate characterizations including HRTEM, AC–HAADF–STEM, EDS and in situ CO–DRIFTS study of the used catalyst don’t show significant structural change compared with the fresh one (Supplementary Figs. 9–10). However, the XRD pattern of used PtCu–SAA catalyst (Supplementary Fig. 9f) displays two weak diffraction peaks centered at 2*θ* 35.5° and 38.7° attributed to the (111) reflection of CuO (JCPDS 048–1548), accounting for the slight deactivation^45^. In order to resolve this issue, the used catalyst was elaborately reduced in a hydrogen stream at 350 °C to induce the reduction of CuO species, followed by another five-cycle catalytic evaluation (Supplementary Fig. 11). Surprisingly, the glycerol conversion rises to 99.2% and the 1,2-PDO selectivity goes up to 98.7% on the sixth recycle; and a slight decrease within 4% occurs during the following four cycles, demonstrating a satisfactory recyclability.

## Discussion

**Fig. 6:**
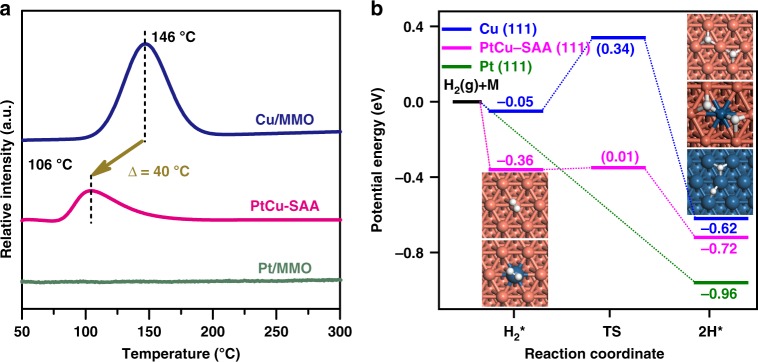
Hydrogen dissociation and spillover capacity. **a** Surface H_2_–TPR spectra of various samples after N_2_O–oxidation pretreatment: Pt/MMO, PtCu–SAA, and Cu/MMO. **b** Potential energies profiles for H_2_ dissociation pathways on the (111) facet of various samples: Pt/MMO (green line), PtCu–SAA (magenta line) and Cu/MMO (blue line), respectively. Numbers in the parentheses represent the reaction barriers of elementary step, and others stand for adsorption energies. H_2_ (g) denotes gas phase of H_2_. Cu, orange, Pt, blue, H, white.

Generally, hydrogen dissociation and spillover capacity over metal catalysts would have an important influence on catalytic performance towards hydrogenolysis reactions. The surface H_2_–TPR measurements after N_2_O pretreatment were performed to study the dissociation and spillover properties of hydrogen on the monometallic Cu/MMO, Pt/MMO, and bimetallic PtCu–SAA catalysts (Fig. 6a). No signal is observed for Pt/MMO sample, implying its weak interaction wtih N_2_O. For Cu/MMO sample, the TPR profile presents a symmetric peak at 146 °C, indicating that the surface Cu_2_O generated through N_2_O pretreatment was reduced by H_2_. As for PtCu–SAA sample, only a broad asymmetrical peak emerges at the region of 106 °C, which is much lower than that of Cu/MMO sample. This sharply decreased temperature and broader signal driven from the introduced atomically dispersed Pt can be ascribed to the enhanced hydrogen dissociation and spillover capacity, respectively^46^. Furthermore, the mechanism of H_2_ adsorption and dissociation was calculated to study the impacts of PtCu alloys containing isolated Pt atoms (Fig. 6b). For PtCu–SAA surface, chemically adsorbed H_2_ is present with an adsorption energy of −0.36 eV (magenta line), indicating H_2_ molecule undergoes a strong adsorption and is easy to be dissociated. As expected, it overcomes an extremely low energy barrier (0.01 eV) to dissociate H_2_ into two H active atoms. In contrast, the hydrogen dissociation is relatively difficult on monometallic Cu surface (with an energy barrier of 0.39 eV, blue line) while it is barrierless on monometallic Pt surface (green line). This indicates that the hydrogen dissociation on PtCu–SAA is between monometallic Cu and Pt, which agrees with the H_2_−TPR results. Therefore, the potential energy barrier of hydrogen dissociation decreases significantly via introducing single Pt atom onto Cu nanoclusters, where dissociated hydrogen atoms would generate spillover from the single metal site and populate the whole Cu surface. This enhanced H_2_ dissociation ability of PtCu–SAA provides abundant hydrogen species required for the carbonyl hydrogenation of intermediate, and thus improves its catalytic activity for hydrogenolysis of glycerol.

Although several studies have been focused on active sites and corresponding reactive pathway for glycerol hydrogenolysis to 1,2-PDO, it is still a great challenge to identify and understand intrinsic active sites of bimetallic alloy structure, owing to the complicated reaction pathway^10,33,47–49^. Herein, the promotional catalytic behavior of PtCu–SAA was revealed via a comprehensive study including in situ glycerol-XAFS, in situ glycerol-DRIFTS experiments and DFT calculations.

**Fig. 7:**
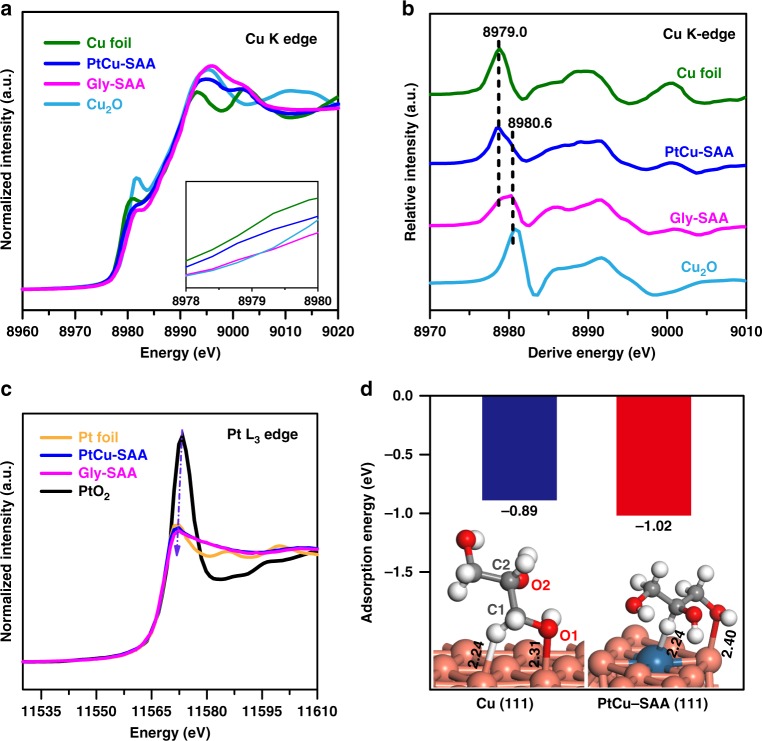
Identification of catalytic active center. **a** In situ normalized XANES spectra and **b** corresponding first derivative profile at Cu K–edge of: Cu foil, Cu_2_O, pristine PtCu–SAA, and PtCu–SAA exposed to glycerol (shorten for Gly–SAA). **c** In situ normalized XANES spectra at Pt L3–edge spectra of Pt foil, PtO_2_, PtCu–SAA, and Gly–SAA. **d** DFT-calculated adsorption energies of glycerol on PtCu–SAA (111) and Cu (111) facet, respectively.

In situ XAFS spectroscopy, which is sensitive for measuring the chemical states of Cu and Pt, was performed to investigate their changes during the catalytic reaction. In situ Cu–K edge XANES spectrum over fresh PtCu–SAA shows a similar absorption edge to Cu foil (Fig. 7a), indicating Cu^0^ is the main chemical state with some Cu^+^ species, in line with the in situ CO–DRIFTS results. Once glycerol is introduced into the PtCu–SAA system, the absorption edge of Cu distinctly shifts to higher energy, which is close to that of Cu^+^ in Cu_2_O. Figure 7b shows the first derivative profiles of XANES spectra with a better sense of chemical states, where the peaks at 8979.0 eV and 8980.6 eV are ascribed to Cu^0^ and Cu^+^, respectively. A drastic shrink occurs for the Cu^0^ peak after the introduction of glycerol, accompanied with a largely increased intensity of Cu^+^ peak. This phenomenon indicates some Cu^0^ species undergoes oxidization to Cu^+^ species. Moreover, the fitting results (Supplementary Table 1) of Fourier transform of *k*^3^-weighted EXAFS spectra (Supplementary Fig. 12) show a governing Cu–Cu coordination (~8.9) at 2.56 Å and another weak Cu–O coordination (~0.8) at 1.86 Å for the fresh PtCu–SAA catalyst. When introducing glycerol, the shell of Cu–Cu and Cu–O remain almost identical; however, the CN of Cu–O increases to 1.6 and Cu–Cu decreases to 4.2, reflecting an evolution of active metal from portion of Cu^0^ to Cu^+^ species^50^. As for the Pt-L3 edge (Fig. 7c), the white line peak displays a slight shift towards low energy compared with the fresh catalyst, suggesting Pt species in PtCu–SAA is reduced to lower valence state in glycerol environment. Thus, in situ glycerol-XAFS results give an experimental evidence that glycerol molecule adsorbs and interacts with surface Pt and Cu species in PtCu–SAA.

Furthermore, we performed DFT calculations to study the nature of active sites and their binding strength to glycerol. The structural models of periodic Cu (111) with/without single Pt atom were constructed to simulate the modified/unmodified Cu nanoclusters, respectively. The adsorption configuration in Fig. 7d shows that the O1 of glycerol is bound to Cu atom (Pt–Cu interface site) with the Cu–O bond length of 2.40 Å, and the C2–H undergoes adsorption on Pt with the formation of Pt–H bond (bond length: 2.24 Å), in agreement with in situ glycerol-XAFS results. Noticed that the adsorption structure of glycerol changes along with Pt addition, which display a lower glycerol adsorption energy on Pt–Cu interface sites (–1.02 eV) compared with Cu–Cu sites (–0.89 eV). As reported previously, the C–H bond breaking plays an important role in determining the glycerol hydrogenolysis^48^. Hence, the formation of Pt–Cu interface changes adsorption sites of glycerol and facilitates C–H bond breaking, resulting in the variation of reaction pathway. Bader charge analysis reveals that electron transfer occurs from H atom to Pt and from Cu to O atom during the activation adsorption of glycerol onto PtCu–SAA surface (Supplementary Fig. 13), consistent with the in situ glycerol-XAFS results. Consequently, the Pt–Cu interface sites of PtCu–SAA serve as the catalytic active center that is responsible for the superior catalytic performance.

**Fig. 8:**
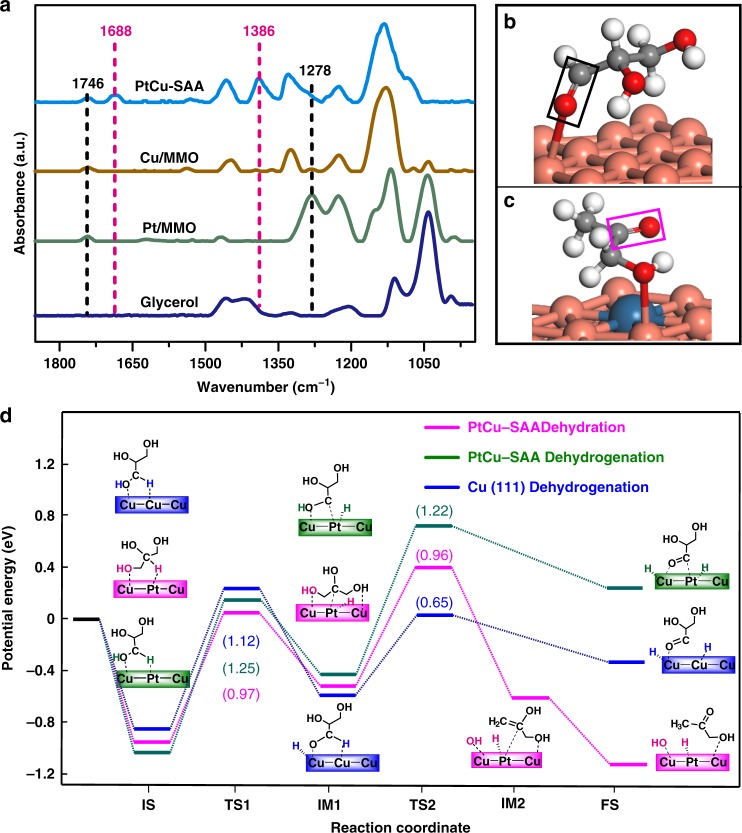
Reaction pathway based on in situ experiments and DFT calculations. **a** In situ DRIFTS spectra of gaseous glycerol and the chemically adsorbed glycerol over Pt/MMO, Cu/MMO, and PtCu–SAA, respectively. The corresponding geometric structures for the consequent products of glycerol dissociation on **b** Cu (111) surface, and **c** PtCu–SAA (111) surface. Cu, orange, Pt, blue, C, grey, O, red, H, white. **d** Potential energy profiles for glycerol hydrogenolysis on Cu (111) and PtCu–SAA (111) surface. The magenta line and green line illustrates dehydration and dehydrogenation step on PtCu–SAA surface, respectively, and the blue line displays dehydrogenation step on monometallic Cu (111) surface. Numbers in the parentheses represent reaction barriers of elementary step.

For purpose of identifying reaction intermediates of glycerol hydrogenolysis, in situ glycerol-DRIFTS measurements were conducted on PtCu–SAA, Pt/MMO, and Cu/MMO catalysts. This validity was confirmed by the gas-phase glycerol hydrogenolysis reaction on PtCu–SAA catalyst, in which catalytic performance in Supplementary Table 6 was obtained compared with previous report^51^. Taking pure gaseous glycerol as reference (Fig. 8a), two strong absorption peaks centered at 1038 and 1111 cm^−1^ are assigned to stretching vibration *ν*(C–O) of primary and secondary C–O bond, and another four peaks centered at 1453, 1413, 1333, and 1209 cm^−1^ are ascribed to *δ*(CH_2_), *δ*(OH), *ρ*(OH), and *ω*(CH_2_), respectively^52^. Compared with the gaseous glycerol spectrum, two clear bands at 1746 and 1278 cm^−1^ are observed on Pt/MMO and Cu/MMO samples (Fig. 8a), which are assigned to *ν*(C=O) and *ν*(O–C–O), respectively. Theses characteristic bands are usually identified as glyceraldehyde originating from the intermediate of glycerol dehydrogenation^53,54^. According to experimental investigations and previous computational studies^4,55^, it is concluded that glycerol undergoes dehydrogenation to glyceraldehyde as the rate-determining step followed by further dehydration and hydrogenation to 1,2-PDO.

In contrast, for PtCu–SAA sample, two strong peaks centered at 1386 and 1688 cm^−1^ appear, which are ascribed to the *ν*(CH_3_) of methyl group and *ν*(C=O) of ketone group, respectively (Fig. 8a)^52^, rather than bending vibration of H_2_O molecule (Supplementary Fig. 14). As suggested previously, glycerol can convert to 2,3-dihydroxypropene via dehydration route; and the subsequent keto-enol tautomerization generates acetol, which serve as two intermediates for the production of 1,2-PDO via hydrogenation process^48^. Combining with in situ glycerol-XAFS and DFT calculations above, this result confirms the formation of acetol species originating from the cleavage of C2–H and C1–O in glycerol. Accordingly, glyceraldehyde and acetol, as two kinds of intermediates derived from glycerol dehydrogenation and dehydration route, can be captured on Cu–Cu and Pt–Cu active sites of PtCu–SAA, respectively, in contrast to the single intermediate (glyceraldehyde) detected on Cu–Cu sites of Cu/MMO. Taking into account the same support and metal particle size between PtCu–SAA and Cu/MMO catalyst, the Pt–Cu interface sites serve as the instinct active centers, in which Pt facilitates activation adsorption of hydrogen atom in secondary carbon (C2–H) while the adjacent Cu accelerates activation adsorption of oxygen atom in primary carbon (C1–O). This interfacial synergistic catalysis can change the reaction path from glycerol dehydrogenation to dehydration and therefore notably boost catalytic activity.

To provide an in-depth understanding for the reaction pathway, DFT calculations were conducted and shown in Fig. 8d. Noted that MgAl–MMO support does not show glycerol hydrogenolysis performance (Supplementary Table 5), suggesting its negligible catalytic effect. Based on the adsorption and activation of reactants, the above intermediates over Cu/MMO, Pt/MMO, and PtCu–SAA catalysts can be reasonably ascribed to metal active sites (Pt–Cu, Cu–Cu, Pt–Pt) rather than MgAl–MMO support. However, these results cannot absolutely exclude the effect of support, since the PtCu–SAA was obtained via the structural transformation from hydrotalcite precursor, with a high dispersion of Cu nanoclusters (Table 1) and good stability (Supplementary Figs. 9–11). The resulting MgAl–MMO support contributes to the formation and loading of Pt–Cu single atom alloy with largely enhanced catalytic efficiency of active site and satisfactory stability. Herein, three possible glycerol hydrogenolysis pathways were investigated, including the dehydration and dehydrogenation on PtCu–SAA interface site, and dehydrogenation on Cu (111) surface. For dehydration on PtCu–SAA interface site, the H of secondary carbon (C2–H) is attacked by Pt site with a barrier of 0.97 eV, followed by the C1–O bond scission on the adjoining Cu site yielding a barrier of 0.96 eV. This pathway produces acetol intermediate (exothermicity: 1.26 eV), in which C2–H bond scission is the rate-determining step. Interestingly, Fig. 8c presents acetol intermediate derived from glycerol dehydration, whose vibration of C=O bond coincides with that of in situ glycerol-DRIFTS spectrum (Fig. 8a). For the dehydrogenation on PtCu–SAA interface site, Pt site catalyzes C1–H bond scission while adjacent Cu site facilitates the cleavage of O1–H bond, resulting in the formation of glyceraldehyde intermediate (endothermicity: 0.15 eV). By comparing the rate-determining step of the dehydration and dehydrogenation, the energy barrier of dehydration path (C2–H scission, 0.97 eV) is much lower than that of dehydrogenation (C1–H scission, 1.25 eV), indicating that glycerol hydrogenolysis is prone to obey dehydration rather than dehydrogenation on PtCu–SAA interface site. Additionally, glycerol dehydrogenation on Cu (111) surface was also investigated, which has been previously reported as the favorable pathway^55^.

As for dehydrogenation on Cu (111), a typical dehydrogenation process occurs on Cu (111) surface: the O–H of primary carbon is attacked by one Cu site yielding a barrier of 1.12 eV, followed by the C1–H bond scission on another Cu site with a barrier of 0.65 eV. This path generates glyceraldehyde intermediate (exothermicity: 0.36 eV), whose O1–H scission is the rate-determining step, in agreement with the in situ glycerol-DRIFTS spectrum of Cu/MMO (Fig. 8a). Making a comparison between dehydration on PtCu–SAA interface site and dehydrogenation on Cu (111), the barrier of rate-determining step in dehydration on PtCu–SAA interface (C2–H scission, 0.97 eV) is lower than that of dehydrogenation on Cu (111) (O1–H scission, 1.12 eV), suggesting that glycerol conversion on PtCu–SAA interface site is more thermodynamically favorable than that on Cu–Cu site. The detailed energies and corresponding geometric structures for the elementary step on different sites are listed in Supplementary Tables 7–9 and Supplementary Fig. 15. This DFT study on reaction mechanism reveals that the introduction of isolated Pt changes reaction pathway with enhanced catalytic reactivity from the thermodynamic point of view.

**Fig. 9:**
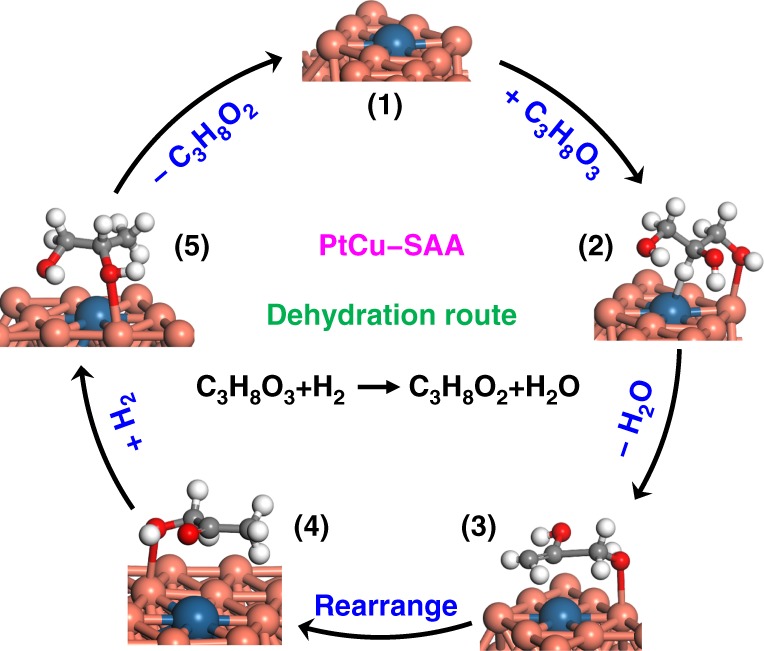
Schematic representation for the reaction mechanism. Schematic illustration for the reaction mechanism of glycerol hydrogenolysis to 1,2-PDO on the surface of PtCu–SAA catalyst.

According to the experimental studies and computational investigations above, a reaction mechanism of glycerol hydrogenolysis over PtCu–SAA catalyst is proposed in Fig. 9. Firstly, glycerol molecule undergoes adsorption and activation on Pt–Cu interface site, in which the O atom (C–O) binding with primary carbon adsorbs on Cu site and the H atom (C–H) at the secondary carbon adsorbs on Pt site. Subsequently, dehydration occurs via cleaving the adsorbed primary C–O and second C–H bond, leading to the formation of a 2,3–enol, followed by a further rearrangement to acetol. Finally, H atom combines with acetol to produce the target product 1,2-PDO. The synergistic catalysis between Cu and isolated Pt, which takes the merits of unique atomic-scale surface active center, is responsible for the significantly improved activity and selectivity.

In summary, we synthesized a PtCu–SAA by utilizing a trace amount of single Pt atom immobilized on Cu derived from an LDHs precursor. In situ CO–DRIFTS, AC–HAADF–STEM and in situ EXAFS results confirm an absolutely isolated Pt atoms dispersed onto the surface of Cu nanoclusters. This as-obtained PtCu–SAA highlights the huge advantage of synergistic catalysis between Cu and Pt, which gives a high catalytic performance and the largest TOF value towards glycerol hydrogenolysis to 1,2-PDO, in comparison with previously reported catalysts. A combination study including in situ experiments and DFT calculations validates that the Pt–Cu interface sites serve as the active center, and the hydrogenolysis reaction follows a low barrier pathway. This interesting finding of interfacial bimetallic synergetic effect at atomic scale provides guidance for rational design and synthesis of bimetallic catalysts via an SAA approach. The method developed in this work can be extended to prepare other high-performance single atom alloy-based catalysts (especially noble metal-involved), with a wide range of heterogeneous catalysis applications.

## Methods

### Chemicals and materials

All reagents (analytical grade) were purchased from Sigma–Aldrich: Mg(NO_3_)_2_·6H_2_O, Cu(NO_3_)_2_·3H_2_O, Al(NO_3_)_3_·9H_2_O, H_2_PtCI_6_·6H_2_O, urea, glycerol, 1,2-propanediol, 1,3-propanediol, ethylene glycol, methanol, ethanol, *n*-propanol, *n*-butanol, acetol. Deionized water was used in all experimental processes.

### Synthesis of catalysts

As precursors, CuMgAl–LDH samples were prepared by a urea hydrolysis and precipitation method. In a typical procedure, a certain amount of Mg (NO_3_)_2_·6H_2_O, Al (NO_3_)_3_·9H_2_O, Cu (NO_3_)_2_·3H_2_O and urea was dissolved in deionized water and sealed in a Teflon-lined stainless-steel autoclave, followed by aging at 110 °C for 24 h. The resulting precipitate was centrifuged, washed with deionized water and dried at 60 °C overnight. The MgAl–LDH samples were synthesized via a similar route without Cu (NO_3_)_2_·3H_2_O. Afterwards, these LDH precursors were calcined in air atmosphere at 500 °C (heating rate: 5 °C∙min^−1^) for 4.0 h, and then cooled down to room temperature to obtained mixed metal oxides (named as MgAl–MMO and CuMgAl–MMO, respectively). Finally, supported Cu samples (named as Cu/MgAl–MMO, shorten for Cu/MMO) were obtained via a reduction treatment of CuMgAl–MMO in a H_2_/N_2_ (1/9, v/v) stream at 350 °C (heating rate: 2 °C ∙min^−1^) for 4.0 h. The supported PtCu SAA and NPs samples were prepared via a galvanic replacement method. In a typical process, the fresh Cu/MMO sample was dispersed in deionized water (100 mL), followed by dropwise adding desired amount of H_2_PtCl_4_ solution (7.7 mM) in N_2_ atmosphere and an ultrasonic ice-water bath for 10 min. The resulting slurry was centrifugated and washed with distilled water, and then dried in a vacuum oven at 60 °C (named as PtCu/MgAl–MMO, shorten for PtCu–SAA). The Pt–Cu alloy samples with various Pt/Cu atomic ratios in a wide range from 0.010 to 0.015, 0.030, 0.15 and 0.50 were denoted as PtCu–SAA–1, PtCu–SAA–2, PtCu–SAA, PtCu–NP–1, and PtCu–NP–2, respectively. As a reference sample, Pt/MMO was prepared by a traditional impregnation method, with identical Pt loading with PtCu–SAA samples.

### Characterizations

The in situ XRD experiments were performed on a Rigaku XRD-6000 diffractometer using Cu Kα radiation (40 kV and 40 mA), which is equipped with the sample cell connected to a gas mass flow and temperature programming device. The chemical composition of the samples was measured with inductively coupled plasma–atomic emission spectrometer (ICP–AES) on the Shimadzu ICPS–7500 device. Multipoint Brunauer–Emmett–Teller (BET) method was adopted to evaluate the total specific surface area using low-temperature N_2_ adsorption–desorption on a Micromeritics ASAP 2020 Instrument. Transmission electron microscopy (TEM) and high-resolution transmission electron microscopy (HRTEM) experiments were carried out using a JEOL JEM–2100 transmission electron microscope. Aberration-corrected high-angle annular dark-field scanning transmission electron microscopy (AC–HAADF–STEM) and element energy dispersive spectroscopy (EDS) mapping images were conducted on a JEOL JEM–ARM200F equipment. In situ EXAFS was performed at the beamline 1W1B of the Beijing Synchrotron Radiation Facility (BSRF), Institute of High Energy Physics (IHEP), Chinese Academy of Sciences (CAS). In situ DRIFTS was carried out on a Bruker TENSOR II equipped with an MCT narrow-band detector.

### Catalytic evaluations for glycerol hydrogenolysis

Glycerol alcoholic solution (10 mL, 10 wt.%) and catalyst at a certain molar ratio were carefully placed into a stainless-steel autoclave reactor (50 mL) at a constant stirring speed of 500 rpm. Afterwards, the reactor was purged with pure H_2_ (99.999%, 2.0 MPa) for five times, and then heated to the certain reaction temperature. After reaction, the resulting products were analyzed on a gas chromatograph (GC–2014C, Shimadzu Company).

### DFT calculations

The periodic DFT calculations were implemented by the Vienna ab initio simulation package (VASP 5.4). General gradient approximation (GGA) of Perdew-Burke-Ernzerhoff (PBE) functional5 was used in all the calculations, and the Grimme’s DFT-D3 method6 was added to investigate the effect of van der Waals interaction. The projector augmented wave (PAW) method was used to describe the core electrons. The energy barriers were determined by the climbing image nudged elastic band (CI-NEB) method.

## Supplementary information

Supplementary Information

## Data Availability

The primary data that support the plots within this paper and other finding of this study are available from the corresponding author on reasonable request.
