# Supplementary material for: Platinum–copper single atom alloy catalysts with high performance towards glycerol hydrogenolysis
Source: Nat Commun. 2019 Dec 20;10:5812. doi: 10.1038/s41467-019-13685-2 (PMC6925196; doi:10.1038/s41467-019-13685-2)
Supplement: Supplementary file 1 — Supplementary Information [file 41467_2019_13685_MOESM1_ESM.pdf]

# Supplementary Information

# Platinum–copper single atom alloy catalysts with high performance towards glycerol hydrogenolysis

Zhang *et al.*

# Supplementary Figures

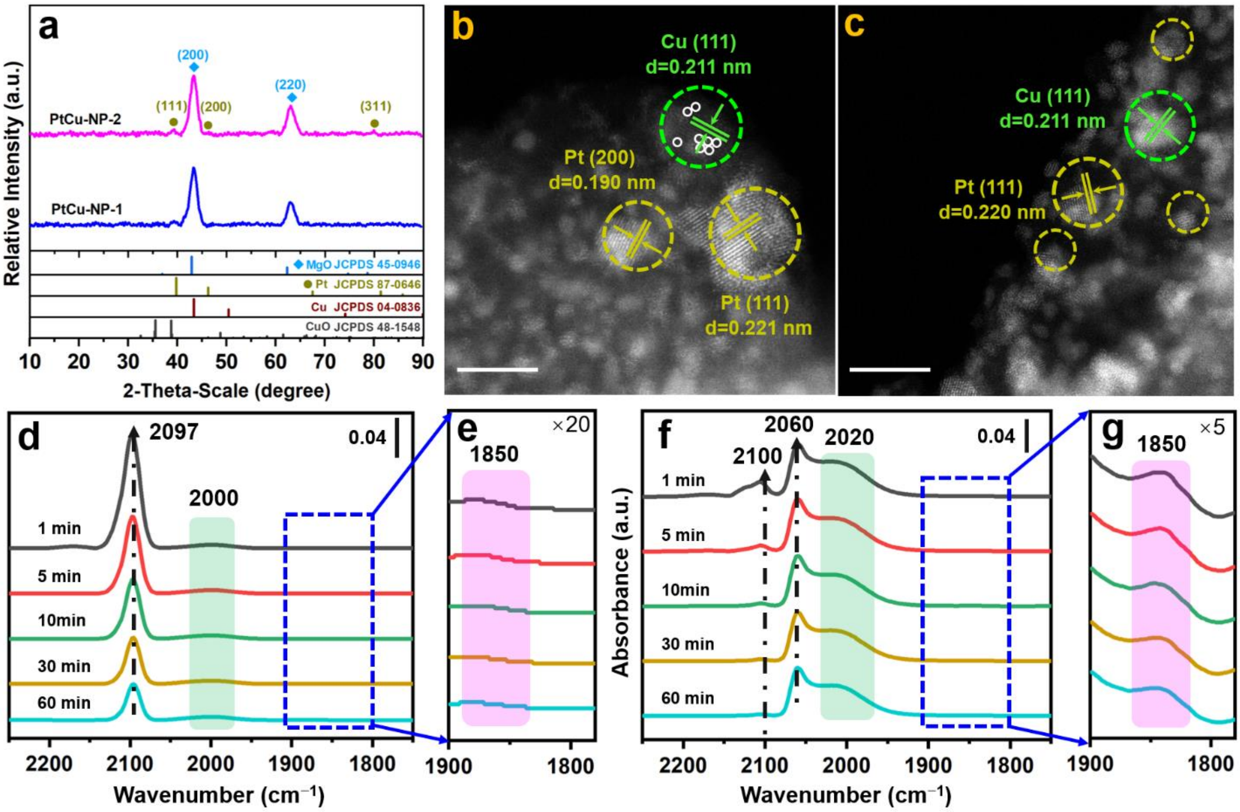


**Supplementary Figure 1 | Structural characterization of PtCu–NPs.** (**a**) The XRD patterns of PtCu–NP−1 and PtCu–NP−2 samples; AC–HAADF–STEM image of (**b**) PtCu–NP−1 and (**c**) PtCu–NP−2; *in situ* CO-DRIFTS spectra of (**d, e**) PtCu–NP−1 and (**f, g**) PtCu–NP−2 sample purging with helium as a function of time. Scale bars: a and b 5 nm

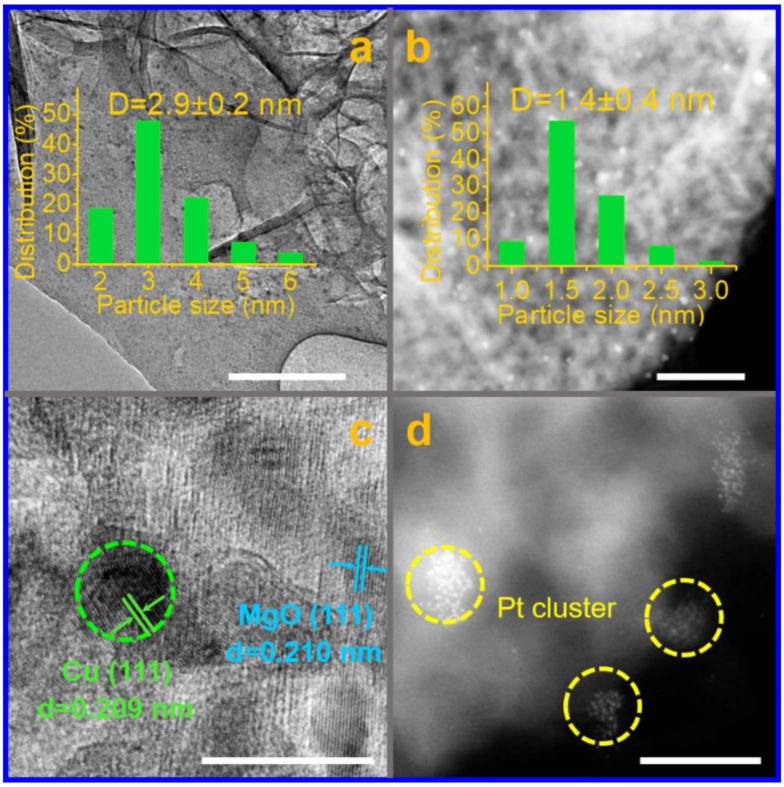


**Supplementary Figure 2 | Structural characterization of Cu/MMO and Pt/MMO.** (**a**) and (**c**): HRTEM images of Cu/MMO; (**b**) and (**d**): AC−HAADF−STEM images of Pt/MMO sample. Scale bars: a 100 nm; b 20 nm; c 10 nm; d 5 nm

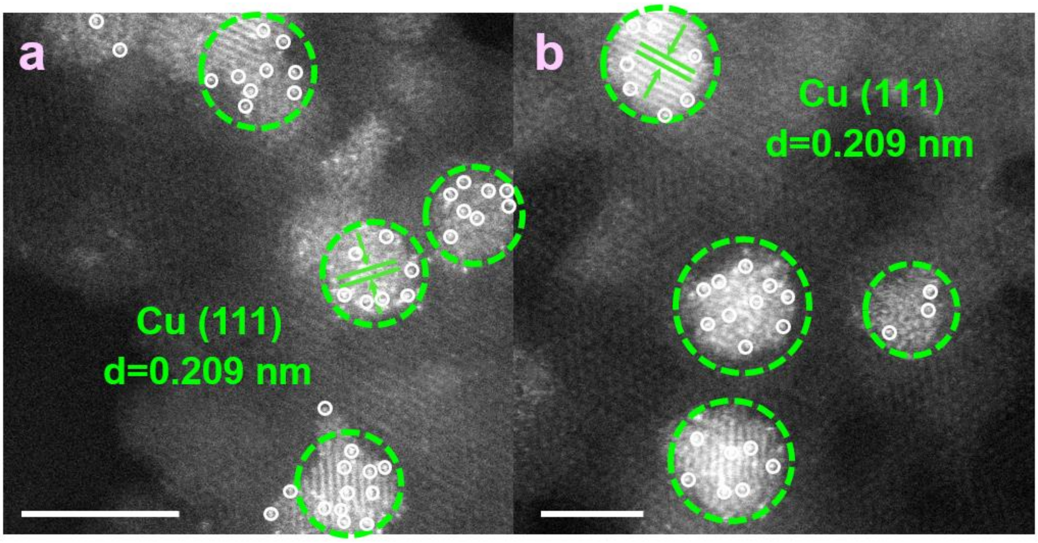


**Supplementary Figure 3 | Structural characterization of PtCu−SAA**. (**a**) and (**b**) The typical AC−HAADF−STEM images of PtCu−SAA sample in different regions; the atomically dispersed Pt atoms (white circles) are clearly observed on the Cu surface. Scale bars: a 5 nm; b 2 nm

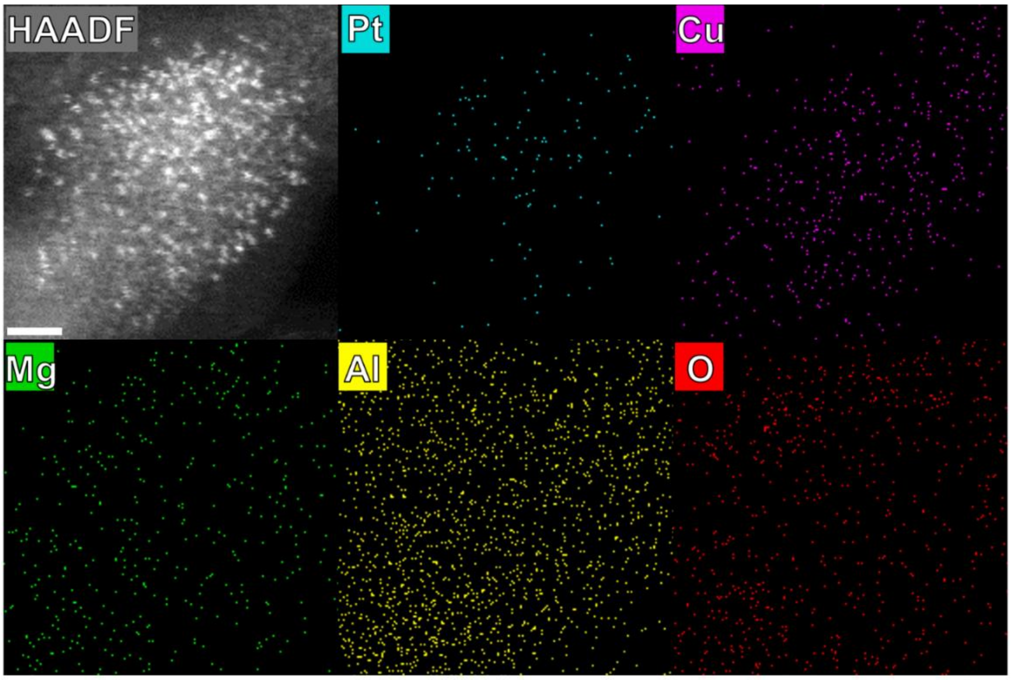


**Supplementary Figure 4 | Element mapping images of PtCu−SAA.** AC−HAADF−STEM images of PtCu−SAA sample and corresponding element EDS mapping of Pt, Cu, Mg, Al and O, respectively. Scale bar: 1 nm

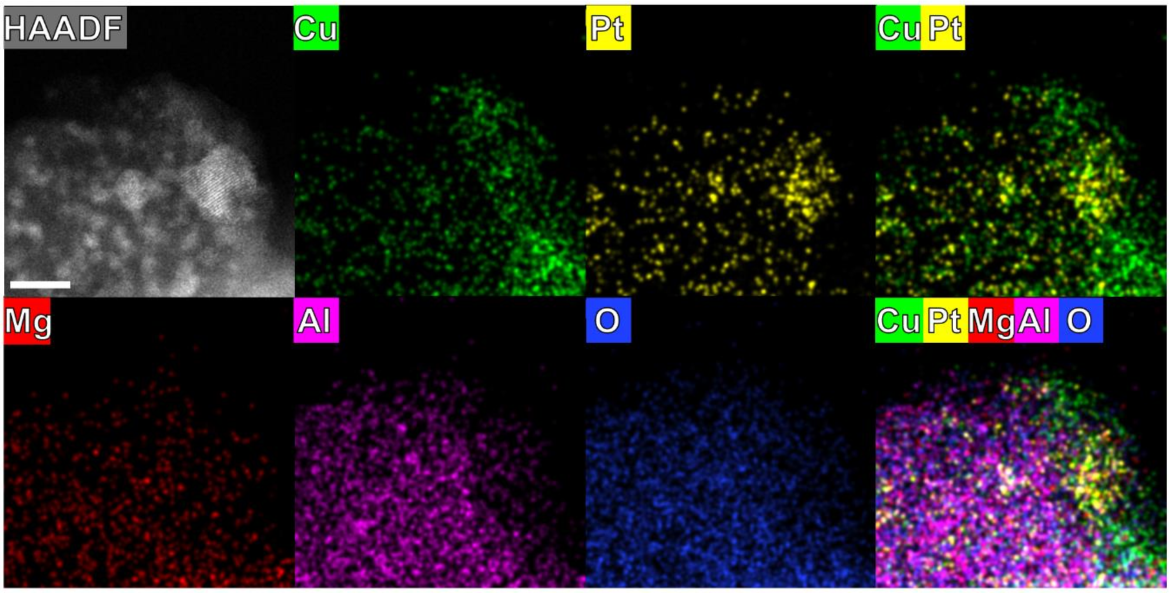


**Supplementary Figure 5 | Element mapping images of PtCu−NP−1 sample.** AC−HAADF−STEM images of PtCu−NP−1 sample and corresponding element EDS mapping of Pt, Cu, Mg, Al and O, respectively. Scale bar: 5 nm

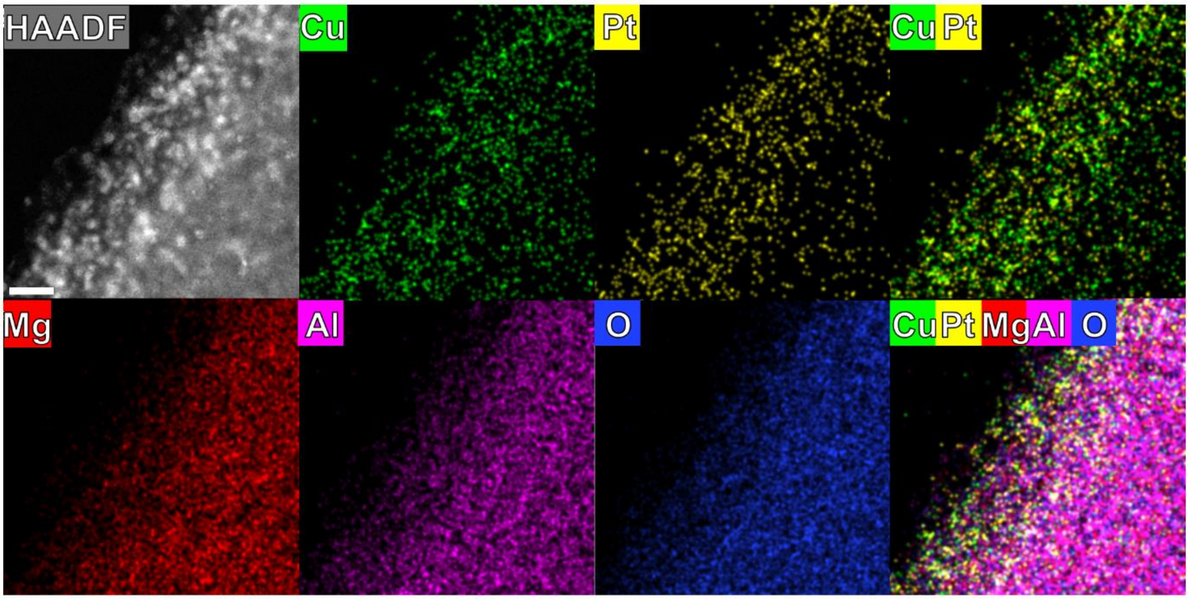


**Supplementary Figure 6 | Element mapping images of PtCu−NP−2 sample.** AC−HAADF−STEM images of PtCu−NP−2 sample and corresponding elemental EDS mapping of Pt, Cu, Mg, Al and O, respectively. Scale bar: 10 nm

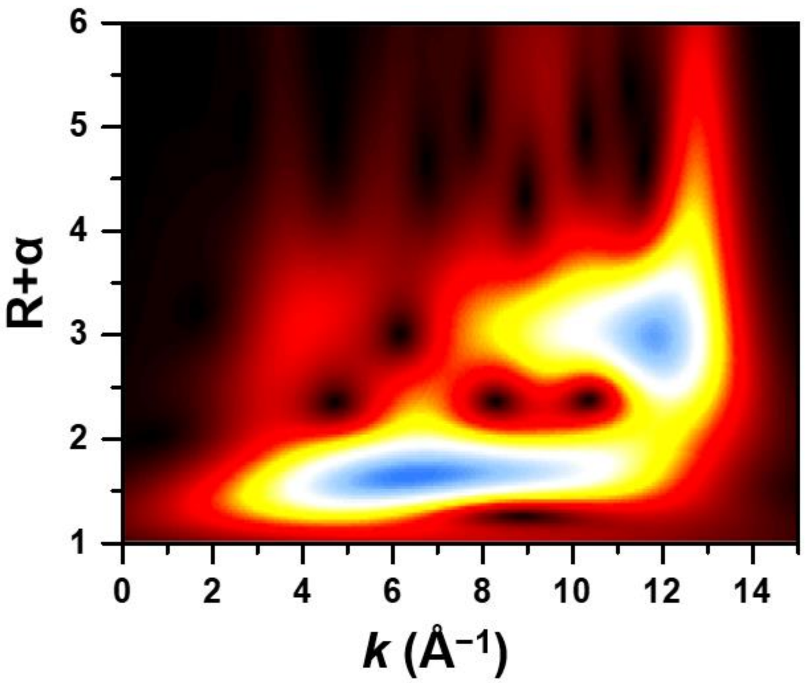


**Supplementary Figure 7 | Structure characterization of $PtO_2$ standard sample.** *In situ* Pt L3–edge EXAFS wavelet transforms spectra of $PtO_2$ standard sample

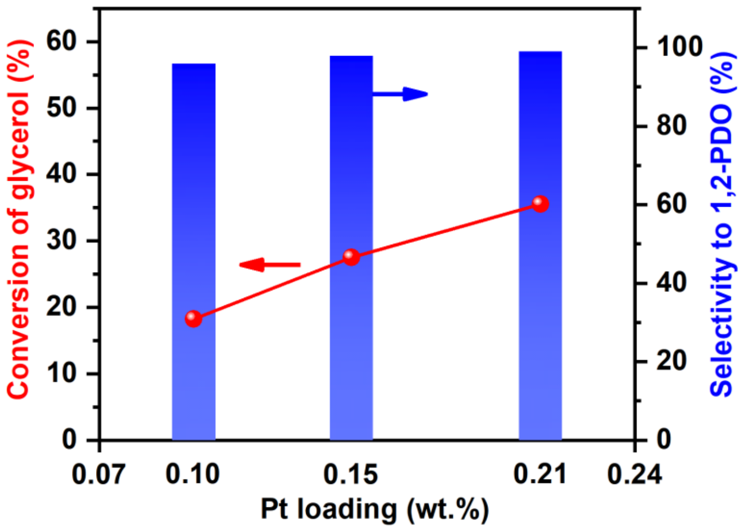


**Supplementary Figure 8 | Identification for the active sites of PtCu−SAA catalyst.** Catalytic evaluations of PtCu–SAA samples with various Pt loading toward glycerol hydrogenolysis to 1,2-PDO. Reaction conditions: 10 mL of glycerol alcoholic solution (0.283 g of glycerol), 0.014 g catalyst, 2.0 MPa of $H_2$ pressure, 200 °C

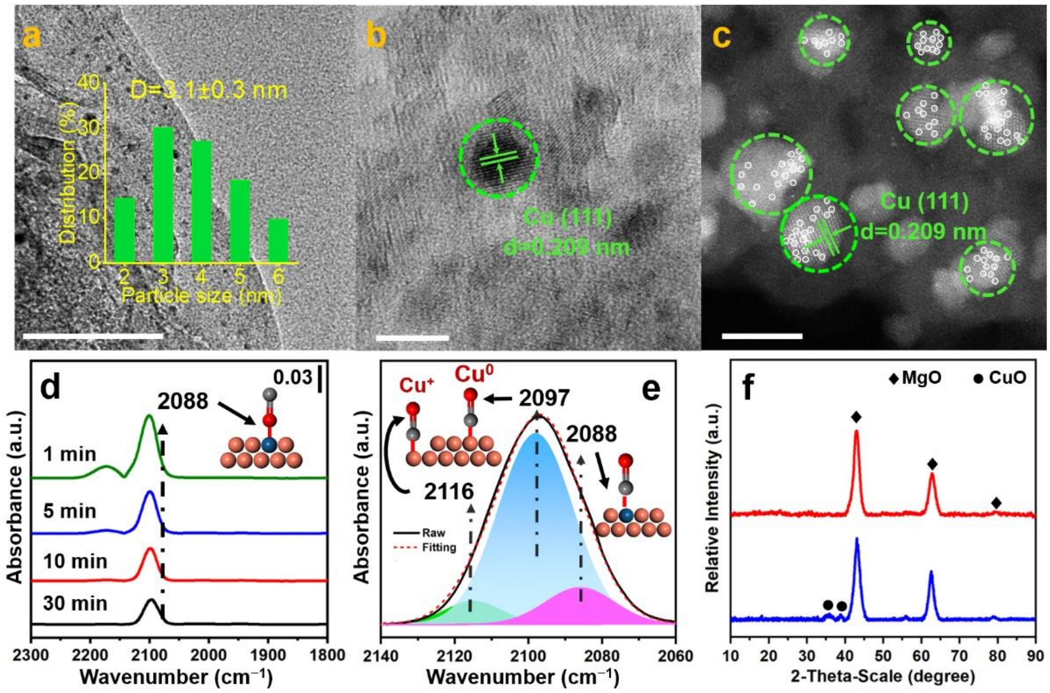


**Supplementary Figure 9. | Characterization of the used PtCu−SAA catalyst.** (**a**) TEM image, (**b**) HRTEM image, (**c**) AC−HAADF−STEM image, (**d**) and (**e**) CO−DRIFTS spectrum of the used PtCu−SAA catalyst after five cycles, (**f**) XRD patterns of the used catalyst (red line) and the regenerated catalyst (blue line). Scale bars: a 100 nm; b and c 5 nm

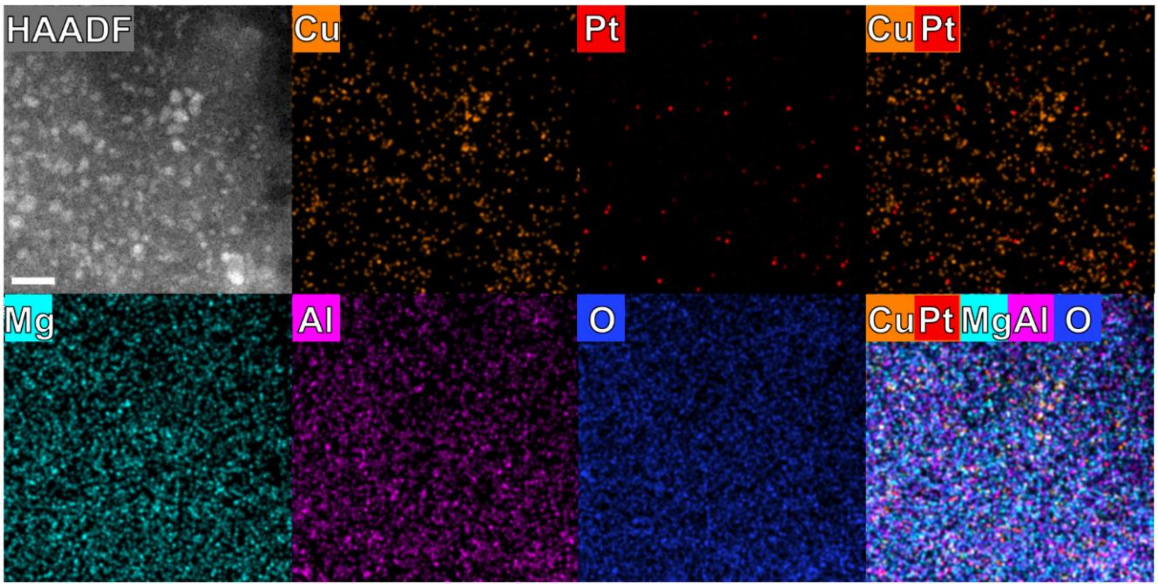


**Supplementary Figure 10 | Element mapping images of the used PtCu−SAA catalyst.** AC−HAADF−STEM images of the used PtCu−SAA catalyst after five cycles and corresponding elemental EDS mapping of Pt, Cu, Mg, Al and O, respectively. Scale bar: 20 nm

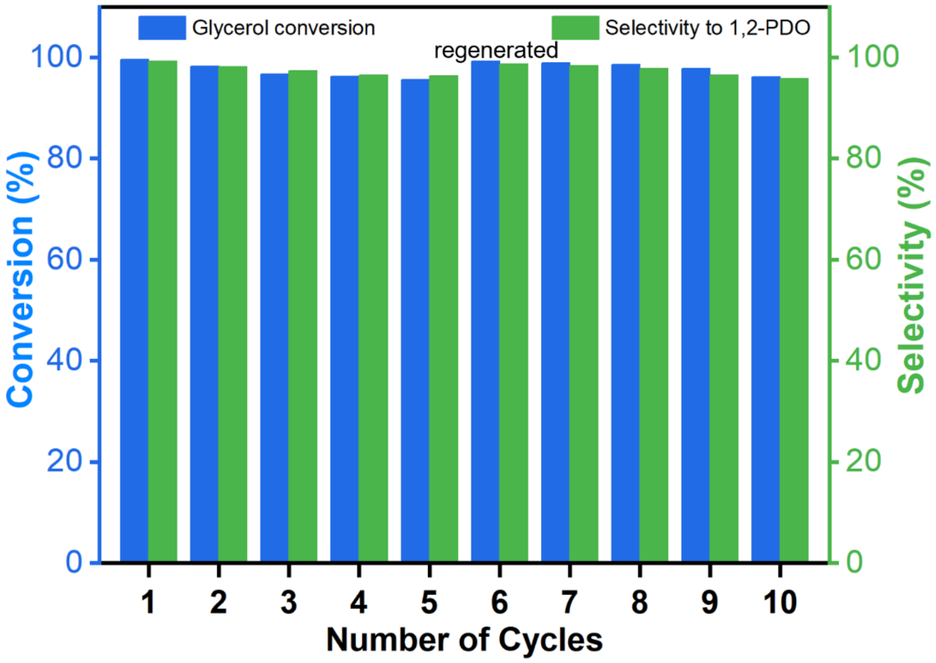


**Supplementary Figure 11 | Catalytic reusability of PtCu–SAA sample.** Catalytic performance of PtCu–SAA catalyst for glycerol hydrogenolysis over five cycles and another five cycles after regeneration

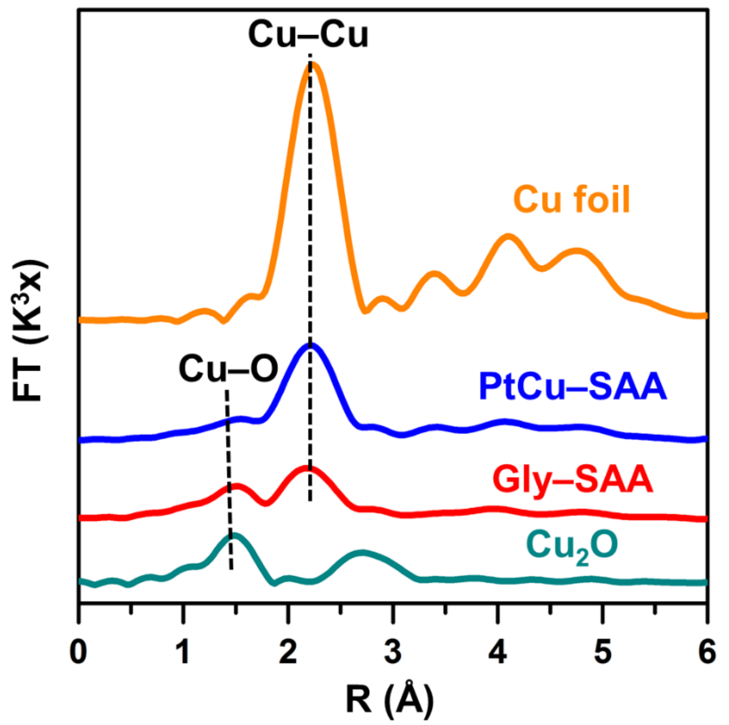


**Supplementary Figure 12 | Structure characterization of various samples at Cu K edge.** *In situ* fourier-transform EXAFS spectra at Cu K–edge for: $Cu_2O$, PtCu–SAA exposed to glycerol (shorten for Gly–SAA), PtCu–SAA, and Cu foil, respectively

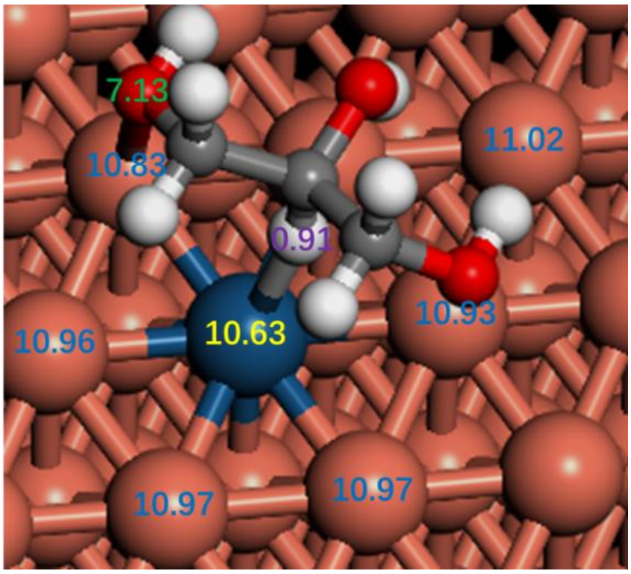


**Supplementary Figure 13 | Surface electronic structure of PtCu−SAA.** Bader charge analysis of glycerol molecule adsorbed on the PtCu−SAA surface

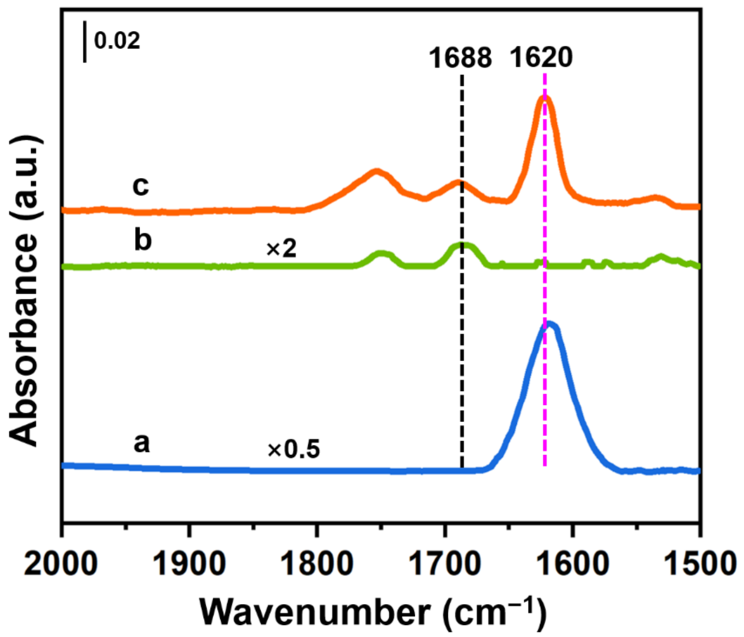


**Supplementary Figure 14 | Determination of the characteristic peak for acetol.** *In situ* DRIFTS spectra of (**a**) water, (**b**) glycerol and (**c**) glycerol aqueous solution in the presence of PtCu–SAA catalyst, respectively

The FTIR spectrum of pure water in the presence of catalyst was measured as a reference sample (Supplementary Figure 14, curve a), which displayed an obvious absorption band centered at about 1620 cm$^{-1}$. This band is usually assigned to the bending vibration of $H_2O$ molecule in agreement with previous report[8]. For purpose of identifying the IR band at around 1688 cm$^{-1}$, *in situ* DRIFTS was conducted on PtCu–SAA by introducing a mixture of water and glycerol as a control sample. Compared with the IR spectrum of pristine gaseous glycerol on PtCu–SAA (Supplementary Figure 14, curve b and c), a new band at about 1620 cm$^{-1}$ is observed, which is assigned to the bending vibration of absorbed $H_2O$ molecule. These comparative experiments verify that the IR band at around 1680 cm$^{-1}$ is not derived from adsorbed water but really from acetol.

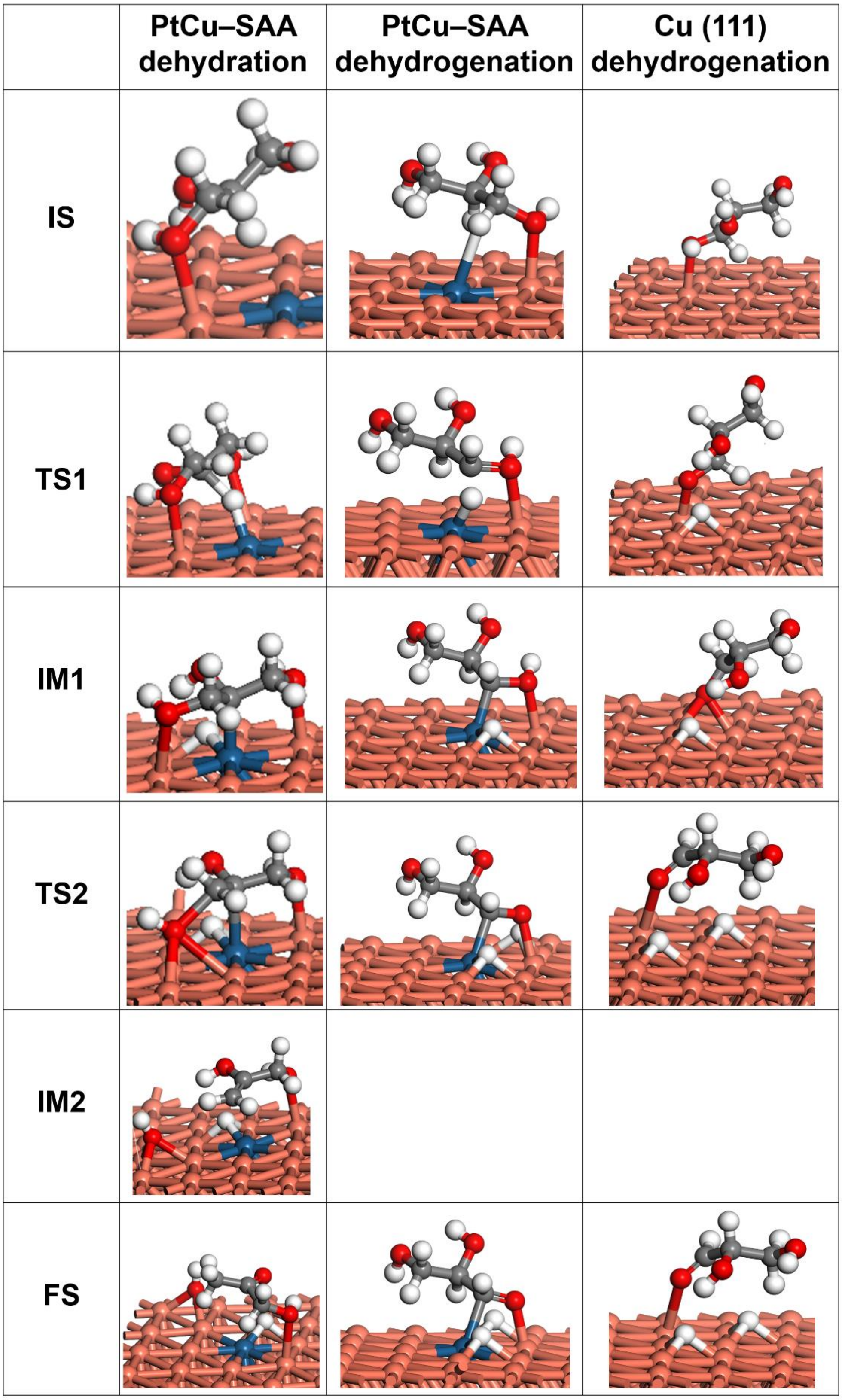


**Supplementary Figure 15 | Geometric structures for the elementary step.** Initial states (IS), transition states (TS), intermediates (IM), and final states (FS) for glycerol dissociation on various pathway of Cu (111) and PtCu−SAA (111) surface, respectively

# Supplementary Tables

**Supplementary Table 1.** EXAFS fitting parameters at the Cu K-edge and Pt $L_3$-edge for various samples

| Sample | Shell | N [a] | R (Å) [b] | $\sigma^2$ ($10^{-3}$ Å$^2$) [c] | $\Delta E_0$ (eV) [d] | R-factor (%) [e] |
|---|---|---|---|---|---|---|
| Pt-foil | Pt−Pt (metallic Pt) | 12 [f] | 2.77(±0.02) | 5.6 | 2.9 | 0.11 |
| $PtO_2$ | Pt−O | 6 | 2.05(±0.01) | 4.4 | −3.6 | 0.24 |
| | Pt−Pt | 6 | 3.09(±0.02) | 5.1 | −6.6 | |
| PtCu-SAA | Pt−Cu (Pt L3) | 7.4 | 2.57(±0.01) | 7.2 | 5.7 | 0.21 |
| PtCu-SAA | Cu−Cu (Cu K) | 8.9 | 2.56(±0.02) | 8.2 | 3.7 | 0.32 |
| | Cu−O (Cu K) | 0.8 | 1.86(±0.03) | 9.4 | −1.8 | |
| Gly-SAA | Cu−Cu (Cu K) | 4.2 | 2.54(±0.02) | 9.2 | 2.4 | 0.41 |
| | Cu−O (Cu K) | 1.6 | 1.87(±0.03) | 6.0 | 3.3 | |
| $Cu_2O$ | Cu−O | 4 | 1.85(±0.01) | 7.6 | 4.7 | 0.27 |
| | Cu−Cu (O-bridged) | 8 | 3.02(±0.01) | 6.7 | 7.7 | |
| Cu-foil | Cu−Cu (metallic Cu) | 12 | 2.56(±0.01) | 5.2 | 5.6 | 0.16 |

[a] *N*: coordination number; [b] *R*: average distance between absorber and backscattered atoms; [c] $\sigma^2$: Debye-Waller factor; [d] $\Delta E_0$: the inner potential correction; [e] *R*-factor: if *r* <5%, consistent with broadly correct models. [f] This value was fixed during EXAFS fitting, based on the known structure of Pt foil. The accuracy of the above parameters was estimated as follows: N, $\sigma^2$, $\Delta E_0$, ± 20%. The data ranges used for fitting in R space ($\Delta R$) are 1.3−3.1 Å.

**Supplementary Table 2.** Catalytic evaluations on Cu/Pt catalysts with various atomic ratios

| Catalyst | Pt/Cu [a] | Pt/Cu [b] | Pt species on Cu surface [c] | Con. | Sel. $_{1,2\text{-PDO}}$ | Sel.$_{\text{Others}}$ |
|---|---|---|---|---|---|---|
| | (mol/mol) | | | (%) | (%) | (%) |
| PtCu−SAA | 0.03 | 0.022 | Single atoms | 99.6 | 99.2 | 0.8 |
| PtCu−NP−1 | 0.15 | 0.12 | Single atoms and clusters | 89.5 | 90.4 | 9.6 |
| PtCu−NP−2 | 0.5 | 0.39 | Clusters and islands | 58.8 | 71.2 | 28.8 |

[a] The nominal atomic ratio. [b] The determined atomic ratio by ICP–AES. [c] Determined by in situ CO-DRIFTS, XAFS, AC−HAADF−STEM and EDS mapping. Reaction conditions: 10 mL of glycerol alcoholic solution (10 wt.%), 0.14 g of catalyst, 2.0 MPa of $H_2$ pressure, 8.0 h, 200 °C.

**Supplementary Table 3.** Comparison studies on catalytic performance toward glycerol hydrogenolysis over various catalysts

| Num. | Catalyst | Tem. | P. | Content [c] | T. | Con. | Sel. | TOF [e] | TOF [f] | TOF [g] | Yield | Pro. [i] | Pro. [j] | Ref. |
|---|---|---|---|---|---|---|---|---|---|---|---|---|---|---|
| | | °C | MPa | wt.% | h | % | % | $h^{-1}$ | $h^{-1}$ | $h^{-1}$ | % | $h^{-1}$ | $h^{-1}$ | |
| 1 | PtCu−SAA [*a*] | 200 | 2 | 0.21; 3.2 [d] | 1 | 11.4 | 98.2 | $2.3\times10^3$<br>$1.2\times10^3$ [h] | 95 | 49 | 11.2 | 890 | 55 | This work |
| 2 | PtCu−SAA [*b*] | 200 | 2 | 0.21; 3.2 [d] | 6 | 99.2 | 99.6 | $5.2\times10^3$<br>$2.6\times10^3$ [h] | $2.1\times10^2$ | $1.1\times10^2$ | 98.8 | 384 | 23.7 | This work |
| 3 | PtCu−SAA [*b*] | 200 | 2 | 0.21; 3.2 [d] | 2 | 82 | 99.6 | | | | 81.7 | 952 | 58.6 | This work |
| 4 | Ru–Cu/$ZrO_2$ | 180 | 2.5 | 2.5; 2.5 [d] | 24 | 13.7 | 100 | 41.4 | 41.4 | 1.5 | 13.7 | 3.1 | 1.6 | 9 |
| 5 | Ru–Cu/$Al_2O_3$ | 200 | 2.5 | 2.5; 2.5 [d] | 24 | 45 | 94 | 32.8 | 32.8 | 7.1 | 42.3 | 9.7 | 4.9 | 9 |
| 6 | Ru–Cu/$Al_2O_3$ | 180 | 2.5 | 2.5; 2.5 [d] | 24 | 20.6 | 91.9 | 11.5 | 11.5 | 2.5 | 18.9 | 4.3 | 2.2 | 9 |
| 7 | Ru–Cu/ MWCNTs | 200 | 4 | 4.8; 28.4 [d] | 6 | 99.8 | 86.5 | 14.0 | 11.8 | 6.5 | 86.3 | 49.5 | 7.2 | 10 |
| 8 | PtRu/C | 200 | 4 | 1.9; 4.7 [d] | 5 | 42 | 24 | 180.0 | 180.0 | 54.0 | 10.1 | 3.4 | 3.4 | 11 |
| 9 | $IrNi_2$ | 200 | 3.6 | 2; 1.2 [d] | 12 | 22.3 | 80.8 | 46.8 | 46.8 | 6.1 | 18.0 | 3.9 | 2.4 | 12 |
| 11 | Ru–Fe/CNT | 200 | 4 | 5 | 12 | 86 | 52.3 | 147.5 | 147.5 | 36.1 | 45.0 | 9.9 | 9.9 | 13 |
| 12 | 5Pt-Re/CNT | 170 | 4 | 5.3 | 8 | 55 | 52 | 51 | 51 | 15.3 | 28.6 | 2.2 | 2.16 | 14 |
| 13 | Ru/$ZrO_2$ | 180 | 2.5 | 2.5 | 24 | 30.1 | 69.8 | 289.8 | 289.8 | 10.1 | 21.0 | 4.8 | 4.8 | 9 |
| 14 | Ru/$Al_2O_3$ | 180 | 2.5 | 2.5 | 24 | 32.8 | 41.7 | 18.4 | 18.4 | 11.4 | 13.7 | 3.1 | 3.1 | 9 |
| 15 | Pt/C | 200 | 4 | 3 | 5 | 13 | 79 | 21.6 | 21.6 | 9.3 | 10.3 | 2.4 | 2.4 | 15 |
| 16 | Ru/MWCNTs | 200 | 4 | 5 | 6 | 65.5 | 71.2 | 198.7 | 198.7 | 53.7 | 46.6 | 25.7 | 25.7 | 10 |
| 17 | Pd/m-$ZrO_2$+ZnO | 220 | 6 | 4.3 | 4 | 40 | 94.1 | 324.7 | 324.7 | 63.6 | 37.6 | 38.9 | 38.9 | 16 |
| 18 | ZnPd/ZnO @$Al_2O_3$ | 230 | 3 | 4.3 | 1.5 | 24.4 | 90.9 | 63.7 | 63.7 | 7.8 | 22.2 | 34.0 | 34.0 | 17 |
| 19 | ZnPd/ZnO | 230 | 3 | 4.3 | 6 | 8.7 | 86.3 | 48.9 | 48.9 | 0.9 | 7.5 | 2.4 | 2.4 | 17 |
| 20 | Cu/MWCNTs | 200 | 4 | 37.6 | 6 | 31.3 | 91.1 | 6.5 | 6.5 | 1.9 | 28.5 | − | 2.1 | 10 |
| 21 | $Cu_{0.4}/Zn_{0.6}Mg_{5.0}Al_2O_{8.6}$ | 200 | 2 | 5.3 | 10 | 85.5 | 98.6 | 18.6 | 18.6 | 9.2 | 84.3 | − | 19.7 | 18 |

[a] Reaction conditions: 10 mL of glycerol alcoholic solution (0.283 g glycerol), 0.014 g of PtCu−SAA catalyst. [b] 10 mL of glycerol alcoholic solution (0.83 g glycerol), 0.14 g of catalyst. [c-d] The content of active metal species ([d] the former is noble metal and the latter means the non-noble metal). [e-h] The TOF value was evaluated by moles of initial glycerol converted per mole of exposed active site atoms per hour (including the surface noble metal atoms [e], the whole surface metal atoms [f], the overall metal atoms [g] and the active sites of SAA atoms [h]). [i-j] The 1,2-PDO productivity (Pro.) was defined as the mass of obtained 1,2-PDO per the overall [j] (noble [i]) metal mass per hour.

**Supplementary Table 4.** Catalytic performance of PtCu−SAA and other reported catalysts at low reaction temperature

| N | Catalyst | T | P | T. | Con | Sel | R1 [a] | R2 [b] | TOF [c] | Pro. [f] | Ref |
|---|---|---|---|---|---|---|---|---|---|---|---|
| | | °C | MPa | h | % | % | mmol·h$^{-1}$·g$^{-1}$-cat | | h$^{-1}$ | h$^{-1}$ | |
| 1 | PtCu−SAA | 120 | 4.0 | 6 | 34 | 97.3 | 2.5 | 2.3 | 2.3×10$^{2}$(Pt) [d]<br>1.1×10$^{2}$(SAA) [e]<br>9.3 (Cu+Pt) [d] | 86.2 | This work |
| 2 | Ru/C+A70($H^+$) | 120 | 8 | 10 | 8 | 68 | 23.1 | 15.7<br>1 | | 24.0 | 19 |
| 3 | Rh-Re$O_x$/$SiO_2$ | 120 | 8 | 5 | 79 | 41 | 47.4 | 18.7<br>8 | | 35.7 | 20 |
| 4 | Ir-Re$O_x$/$SiO_2$ | 120 | 8 | 1 | 7 | 9;69 [g] | 50.7 | 4.6 | 284 [d] | 34.7 | 21 |
| 5 | Ir-Re$O_x$/$SiO_2$ | 120 | 8 | 1 | 38 | 7;58 [g] | 69.4 | 4.86 | − | 14.7 | 21 |
| 6 | Pt-Re/$SiO_2$ | 120 | 4 | 1 | 8.3 | 40 | 5.1 | 2.03 | 32.5 [d] | 1.93 | 22 |
| 7 | Pt/SBA-15 | 150 | 4 | 30 | 4 | 10.6 | 0.04 | 0.00<br>5 | 2.8 [d] | 0.01 | 23 |
| 8 | CuO/$SiO_2$-PG | 160 | 9 | 12 | 19 | 98 | 2.8 | 2.70 | 1.9 [d] | 0.7 | 24 |
| 9 | CuO/$SiO_2$-IM | 160 | 9 | 12 | 2 | 99 | 0.3 | 0.29 | 1.0 [d] | 0.07 | 24 |
| 10 | Cu/3Ce/Mg | 160 | 6 | 10 | 19 | 94 | 2.0 | 1.86 | 14.6 [d] | 1.8 | 25 |

Reaction conditions: 10 mL of glycerol alcoholic solution (10 wt.%), 0.21 g of catalyst. [a] The average reaction rate of glycerol conversion based on the mol of glycerol converted per catalysts mass and per hour. [b] The average formation rate of 1,2-PDO based on the mol of 1,2-PDO obtained per catalysts mass and per hour. [c-e] The TOF value was evaluated by the number of glycerol converted per hour per catalytic site (including of the surface metal atoms [d] and the active sites atoms [e]). [f] The 1,2-PDO productivity (Pro.) was defined as the mass of obtained 1,2-PDO per the overall active metal mass per hour. [g] The former is the selectivity of 1,2-PDO while the latter means that of 1,3-propanediol.

As shown in Supplementary Table 4, the PtCu−SAA catalyst displays a rather low glycerol conversion and TOF value at 120 °C than that at 200 °C; while the selectivity of 1,2-PDO maintains ~97.3% at 120 °C, close to 99.2% at 200 °C. The average reaction rate of glycerol and TOF value based on surface (active sites) metal atom of PtCu−SAA is lower than that of 4%Rh-Re$O_x$ and 20%Ir-Re$O_x$ catalysts (entry 3−5) at 120 °C and 8.0 MPa. It should be noted that the catalytic performance, TOF value and the 1,2-PDO productivity based on the overall active metal of PtCu−SAA are much higher than most Pt and/or Cu based catalysts at ~120 °C.

**Supplementary Table 5.** Catalytic performance toward glycerol hydrogenolysis over MgAl−MMO sample

| Catalyst | Tem. (°C) | P. (MPa) | Time (h) | $Con._{Gly}$ (%) |
|---|---|---|---|---|
| MgAl−MMO | 200 | 2.0 | 8.0 | 1.3 |

Reaction conditions: 10 mL of glycerol alcoholic solution (10 wt. %), 0.2 g of catalyst.

**Supplementary Table 6.** Catalytic performance of PtCu−SAA catalyst by using a fixed bed

| Number | Catalyst | Gas | $Con._{GLY}$ (%) | $Sel._{1,2\text{-}PDO}$ (%) | $Sel._{acetol}$ (%) | $Sel._{others}$ (%) |
|---|---|---|---|---|---|---|
| 1 | PtCu−SAA | $H_2$ | 12.5 | 15.6 | 79.7 | 4.7 |
| 2 | PtCu−SAA | $N_2$ | 10.2 | 1.8 | 92.4 | 5.8 |

Reaction conditions: Temperature: 200 °C; Pressure: 0.1 MPa; WHSV: 2.0 $g \cdot g_{cat}^{-1} \cdot h^{-1}$; catalysts: 50 mg; $H_2$/Gly molar ratio: 80.

**Supplementary Table 7.** Born–Oppenheimer electronic energies for PtCu–SAA *via* dehydration pathway

| Reaction | Δ*E* (eV) |
|---|---|
| $CH_2OH$-CHOH-$CH_2OH$ (g) + * → $CH_2OH$-CHOH-$CH_2OH$* (1) | −1.02 |
| $CH_2OH$-CHOH-$CH_2OH$* (1) → $CH_2OH$-COH-$CH_2OH$* + H* (2) | 0.47 |
| $CH_2OH$-COH-$CH_2OH$* + H* (2) → $CH_2OH$-COH-$CH_2$* + H* + OH* (3) | −0.18 |
| $CH_2OH$-COH-$CH_2$* + H* + OH* (3) → $CH_2OH$-CO-$CH_3$* + H* + OH* (4) | −0.53 |

**Supplementary Table 8.** Born–Oppenheimer electronic energies for PtCu–SAA *via* dehydrogenation pathway

| Reaction | ΔE (eV) |
|---|---|
| $CH_2OH$-CHOH-$CH_2OH$ (g) + * → $CH_2OH$-CHOH-$CH_2OH$* (1) | −1.09 |
| $CH_2OH$-CHOH-$CH_2OH$* (1) → $CH_2OH$-COH-$CH_2OH$* + H* (2) | 0.62 |
| CH2OH-COH-CH2OH* + H* (2) → CH2OH-COH-CH2O* + 2H* (3) | 0.62 |

**Supplementary Table 9.** Born–Oppenheimer electronic energies for Cu (111) *via* dehydrogenation pathway

| Reaction | ΔE (eV) |
|---|---|
| $CH_2OH$-CHOH-$CH_2OH$ (g) + * → $CH_2OH$-CHOH-$CH_2OH$* (1) | −0.89 |
| $CH_2OH$-CHOH-$CH_2OH$* (1) → $CH_2OH$-CHOH-$CH_2O$* + H* (2) | 0.28 |
| $CH_2OH$-CHOH-$CH_2O$* + H* (2) → $CH_2OH$-COH-$CH_2O$* + 2H* (3) | 0.25 |

# Supplementary Methods

## The details of galvanic replacement method.

As the standard reduction potential of $PtCl_6^{2-}/Pt$ redox pair (1.44 V *vs.* the standard hydrogen electrode (SHE)) is much higher than that of $Cu^{2+}/Cu$ redox pair (0.34 V *vs.* SHE), the following thermodynamically-favorable redox reaction occurs:

$$2Cu(s) + PtCl_6^{2-}(aq) = Pt(s) + 2Cu^{2+}(aq) + 6Cl^{-}(aq) \quad (1)$$

The large potential difference (up to 0.76 V) guarantees the reaction feasibility and makes this replacement reaction occur rapidly at room temperature. Moreover, Muhler *et al.*[1] have addressed how this thermodynamically-favorable reaction occurs at $T$ = 0 °C.

## *In situ* XRD experiments

The as-obtained sample was elaborately reduced in a $H_2/N_2$ (1/9, v/v) stream at 350 °C (heating rate: 2 °C ·min$^{-1}$) for 4.0 h, followed by flushing (100 mL ·min$^{-1}$) with high purity $N_2$ for 1.0 h. The reduced sample was slowly cooled down to room temperature in a $N_2$ atmosphere, and then the XRD patterns were precisely recorded with a 2$\theta$ scan range from 5° and 90° at a low scanning rate of 5°·min$^{-1}$.

## Surface $H_2$–TPR experiments

Surface hydrogen temperature programmed reduction ($H_2$−TPR) after the $N_2O$−oxidation treatment was measured on a Micromeritics Chemi-Sorb 2920 equipped with a thermal conductivity detector (TCD). Typically, the sample (50 mg) was pre−reduced in a $H_2$/Ar (1/9, v/v, 100 mL·min$^{-1}$) atmosphere at a rate of 5 °C·min$^{-1}$ from room temperature to 350 °C, and then cooled down to 50 °C in an Ar flow (50 mL·min$^{-1}$). Subsequently, the $N_2O$/Ar (1/9, v/v, 50 mL·min$^{-1}$) was introduced

to oxidize the sample for 2.0 h. And then the sample was heated to 150 °C at 5 °C·min$^{-1}$ and hold at 150 °C for 1.0 h in an Ar flow (50 mL·min$^{-1}$) to release residual $N_2O$. Finally, the sample was slowly cooled down from 150 °C to 50 °C, followed by purging $H_2$/Ar (1/9, v/v, 50 mL·min$^{-1}$) along with increasing temperature to 350 °C at a rate of 5 °C·min$^{-1}$. Meanwhile, the surface $H_2$−TPR profile was recorded by a TCD detector.

**$N_2O$ chemisorption experiments**

The dispersion of Cu and specific surface area of metallic Cu were determined by $N_2O$ chemisorption and hydrogen pulse reduction method on a Micromeritics Autochem II 2920 equipped with a TCD. Briefly, after the pretreatment with He flow at 200 °C for 2.0 h, the sample (100 mg) was reduced in a $H_2$/Ar flow (1/9, v/v; 50 mL·min$^{-1}$) at 300 °C for 2 h, and the corresponding hydrogen consumption (denoted as *X*) was detected. Afterwards, the sample was flushed with high purity Ar for 1.0 h and cooled down to 150 °C. Subsequently, a pure flow of $N_2O$ was induced at a rate of 50 mL·min$^{-1}$ for 2.0 h, guaranteeing a complete oxidization of surface Cu atoms to $Cu_2O$. Then successive pulses of high-purity $H_2$ were introduced at 300 °C to reduce surface $Cu_2O$ to metallic Cu, with Ar as carrier gas (50 mL·min$^{-1}$). The volume of consumed hydrogen was obtained by subtracting the sum of residual unsaturated hydrogen pulses from the total hydrogen injection volume (denoted as *Y*). The dispersion of metallic Cu (*D*%, eq. 1), was obtained according to $N_2O$ chemisorption value:

$$D(\%) = \frac{2\times \mathrm{Y}}{X} \times 100 \quad (2)$$

**Hydrogen oxygen ($H_2$−$O_2$) titration experiments**

The Hydrogen-oxygen titration (HOT) experiments were carried out on a Micrometric Autochem II 2920 equipped with a thermal conductivity detector (TCD). Briefly, after the

pretreatment in a $H_2$/Ar flow (1/9, v/v; 50 mL·min$^{-1}$) at 300 °C for 2 h, the sample (100 mg) was exposed to a flow of pure Ar (50 mL·min$^{-1}$) until the baseline was stabilized at 150 °C. Subsequently, the $O_2$/Ar (1/9, v/v) pulses were introduced into the carrier gas (50 mL·min$^{-1}$) until total saturation was reached to 150 °C. Subsequently, the absorbed oxygen was titrated by introducing pulses of $H_2$/Ar flow (1/9, v/v) at 150 °C. Finally, the consumption of $H_2$ was monitored and recorded by the TCD detector. The Pt dispersion was calculated based on the relation of HT: OT: Pt = 1.5:0.75:1.

***In situ* XAS characterization**

*In situ* extended X-ray absorption fine structure spectroscopy (EXAFS) at the Cu *K*-edge and Pt $L_3$-edge was carried out at the beamline 1W1B of the Beijing Synchrotron Radiation Facility (BSRF), Institute of High Energy Physics (IHEP), Chinese Academy of Sciences (CAS). The powdered sample was pressed into a thin round sheet with a diameter of 13 mm and carefully placed into a reaction microdevice equipped with polyimide windows. The detailed experiment procedure is described as follows: The Cu/MMO, CuPt-SAA and Pt/MMO were pre-reduced in a $H_2$/He flow (1/9, v/v; 50 mL·min$^{-1}$) at 350 °C, followed by purging He (50 mL·min$^{-1}$) for 1.0 h and then cooled to room temperature in a He stream. Finally, the EXAFS spectra of Cu *K*-edge or Pt $L_3$-edge was collected as *in situ* EXAFS results. As for the *in situ* glycerol-XAFS, based on the above pretreatment, a certain amount of glycerol was carefully evaporated and pulsed into the thin cell in He flow (50 mL·min$^{-1}$) for 10 min, followed by evacuation to remove weakly-adsorbed glycerol, and then XAFS spectra were collected until a signal was obtained. Data processing and analysis were performed using the IFEFFIT package[2]. The amplitude reduction factor ($S_0^2$) was obtained from the fitting results of experimental EXAFS data of Pt foil as follows[2–5]: the coordination number (CN) of experimental Pt foil was fixed during EXAFS fitting process leading to the $S_0^2$ value, based

on the known crystallographic structure value of Pt foil. And then, this $S_0^2$ value was fixed to all the samples.

### *In situ* DRIFTS experiments

*In situ* diffuse reflectance Fourier transform infrared spectroscopy (*in situ* DRIFTS) of CO and glycerol was performed on a TENSOR 27 (Bruker Company) spectrometer equipped with an MCT narrow-band detector and a modified *in situ* reaction cell with a drier device. The detailed pre-treatment and test conditions are given as follows. Firstly, about 50 mg of sample was carefully put into the support sheet of reaction cell. Afterwards, the sample was pre-reduced in a $H_2$/He flow (1/9, v/v; 50 mL $\cdot$min$^{-1}$) at 350 °C with a heating rate of 5 °C min$^{-1}$, followed by purging He (50 mL $\cdot$min$^{-1}$) for 1.0 h and cooled to 25 °C in a pure He stream. In this process, the background spectrum was collected at different temperatures (25, 50, 80, 100 °C). Subsequently, the CO/He or glycerol/He (1/19, v/v; 50 mL $\cdot$min$^{-1}$) was introduced into the cell, and then DRIFTS spectra were collected until CO or glycerol/He adsorption signals kept unchanged. Finally, the gas flow was switched to a high purity He stream, along with an increase of temperatures (25, 50, 80, 100 °C) to collect desorption spectra.

### Computational section

The periodic DFT calculations were implemented by the Vienna ab initio simulation package (VASP 5.4). General gradient approximation (GGA) of Perdew-Burke-Ernzerhoff (PBE) functional was used to solve Kohn-Sham equation, and the Grimme's DFT-D3 method was added to investigate the effect of van der Waals interaction[6]. The projector augmented wave (PAW) method was used to describe the core electrons. A 3 × 3 × 1 Monkhorst−Pack $k$ point mesh and a 400 eV cutoff energy

for the plane-wave basis were employed for the geometry optimizations. The convergence criterion for the total energy self-consistent iterations was $10^{-4}$ eV, and the geometry optimization stops when the total force on the system was less than 0.02 eV/Å. The energy barriers were determined by the climbing image nudged elastic band (CI-NEB) method[7].

According to the HRTEM characterization, the Cu (111) surface was modeled with a four-layer-slab in a $p(4\times4)$ surface unit cell; the bottom two slabs are fixed while the top two slabs are relaxed. A vacuum space of 15 Å ensures no spurious interaction between the periodically repeated slabs or adsorbates in the direction normal to the slab. The CuPt (111) surface was built by substituting one Cu atom on the topmost layer with one Pt atom. It should be noted that all the samples (Cu/MMO, PtCu–SAA and Pt/MMO) were calculated based on the metal surface rather than the interface model, since they have the same mixed metal oxides support (MgO and $Al_2O_3$) and the effect of support can be neglected in calculating the relative energies (such as adsorption energy and reaction barrier).

The adsorption energies ($E_{ads}$) of reactants/products were obtained based on the equation:

$$E_{ads}=E_{total}-(E_{surf}+E_{adasorbate}) \quad (3)$$

where $E_{total}$, $E_{surf}$ and $E_{adasorbate}$ represent the energy after adsorption, clean surface and free adsorbate (gas phase), respectively.

**Catalytic Testing**

Glycerol alcoholic solution (10 mL, 10 wt.%) and catalyst at a certain molar ratio were was carefully placed into a stainless steel autoclave reactor (50 mL) at a constant stirring speed of 500 rpm. Afterwards, the reactor was purged with pure $H_2$ (99.999%, 2.0 MPa) for five times, and then heated to the reaction temperature (200 °C). After the reaction, the autoclave was placed in an ice water bath, and the pressure was released carefully. The obtained liquid-phase products were

separated by filtration and analyzed using a DB−WAX−UI capillary column (Agilent Technologies Company, 30m×0.25mm×0.25mm) and a flame ionization detector (FID). In addition, few of fresh catalysts were compensated to achieve the same ratio between glycerol and catalysts, due to the slight loss of used catalysts during the recycle test process. The conversion of glycerol and the selectivity of products were calculated by following equations:

$$X_{Gly}=\frac{[\mathrm{Glycerol}]_{\mathrm{in}}-[\mathrm{Glycerol}]_{\mathrm{out}}}{[\mathrm{Glycerol}]_{\mathrm{in}}}\times 100\% \quad (4)$$

$$\mathrm{Selectivity}(\%)=\frac{Molar\ amount\ of\ carbon\ in\ specific\ product}{Total\ molar\ amount\ of\ carbon\ in\ converted\ glycerol}\times 100\% \quad (5)$$

The turnover frequency (TOF) was measured as moles of initial glycerol converted per mole of exposed active site atoms per hour ($mol_{glycerol}\cdot mol_{M}^{-1}\cdot h^{-1}$, M represent surface metal). It should be noted that the mole of exposed active site atoms of the PtCu−SAA catalysts was calculated based on Pt-Cu single atom alloy sites with two atoms while those of Cu/MMO and Pt/MMO catalysts was evaluated by the exposed surface metal atoms.

$$\mathrm{TOF}=\frac{k\times Molar\ amount\ of\ initial\ glycerol}{Molar\ amount\ of\ exposed\ ative\ site\ atoms}\times 100\% \quad (6)$$

The *k* means the calculated reaction rate constant from Fig. 5A and B.

**Supplementary References**


1. Sun, Z. et al. Rapid and surfactant-free synthesis of bimetallic Pt–Cu nanoparticles simply via ultrasound-assisted redox replacement. *ACS Catal.* **2**, 1647–1653 (2012).
2. Newville, M. IFEFFIT: interactive XAFS analysis and FEFF fitting. *J. Synchrotron Radiat.* **8**, 322–324 (2001).
3. Zabinsky, S. I., Rehr, J. J., Ankudinov, A., Albers, R. C. & Eller, M. J. Multiple-scattering calculations of X-ray-absorption spectra. *Phys. Rev. B* **52**, 2995–3009 (1995).
4. Benfield, R. E. Mean coordination numbers and the non-metal-metal transition in clusters. *J. Chem. Soc. Faraday Trans.* **88**, 1107–1110 (1992).
5. Sasaki, K. & Marinkovic, N. in *X-Ray and Neutron Techniques for Nanomaterial Characterization* (ed. Kumar, C. S. S. R.) Ch. 6 (Springer, 2016).
6. Henkelman, G., Uberuaga, B. P. & Jónsson, H. A climbing image nudged elastic band method for finding saddle points and minimum energy paths. *J. Chem. Phys.* **113**, 9901–9904 (2000).
7. Perdew, J. P., Burke, K. & Ernzerhof, M. Generalized gradient approximation made simple. *Phys. Rev. Lett.* **77**, 3865–3868 (1996).
8. Paze, C., Bordiga, S. & Zecchina, A. $H_2O$ interaction with solid $H_3PW_{12}O_{40}$: an IR study. *Langmuir* **16**, 8139–8144 (2000).
9. Soares, A. V. H. et al. A study of glycerol hydrogenolysis over Ru–Cu/$Al_2O_3$ and Ru–Cu/$ZrO_2$ catalysts. *J. Mol. Catal. A: Chem.* **415**, 27–36 (2016).
10. Wu, Z., Mao, Y., Wang, X. & Zhang, M. Preparation of a Cu–Ru/carbon nanotube catalyst for hydrogenolysis of glycerol to 1,2-propanediol *via* hydrogen spillover. *Green Chem.* **13**, 1311–1316 (2011).
11. Maris, E. P., Ketchie, W. C., Murayama, M. & Davis, R. J. Glycerol hydrogenolysis on carbon–supported PtRu and AuRu bimetallic catalysts. *J. Catal.* **251**, 281–294 (2007).
12. Pamphile–Adrián, A. J., Florez–Rodriguez, P. P., Pires, M. H. M., Perez, G. & Passos, F. B. Selective hydrogenolysis of glycerol over Ir–Ni bimetallic catalysts. *Catal.Today* **289**, 302–308 (2017).
13. Li, B. et al. Carbon nanotube–supported RuFe bimetallic nanoparticles as efficient and robust catalysts for aqueous–phase selective hydrogenolysis of glycerol to glycols. *ACS Catal.* **1**, 1521–1528 (2011).
14. Deng, C. et al. Size effects of Pt-Re bimetallic catalysts for glycerol hydrogenolysis. *Catal.Today* **234**, 208–214 (2014).

15. Maris, E. P. & Davis, R. J. Hydrogenolysis of glycerol over carbon–supported Ru and Pt catalysts. *J. Catal.* **249**, 328–337 (2007).
16. Sun, Q., Wang, S. & Liu, H. Selective hydrogenolysis of glycerol to propylene glycol on supported Pd catalysts: promoting effects of ZnO and mechanistic assessment of active PdZn alloy surfaces. *ACS Catal.* **7**, 4265–4275 (2017).
17. Li, X. et al. A facile strategy for confining ZnPd nanoparticles into a ZnO@$Al_2O_3$ support: a stable catalyst for glycerol hydrogenolysis. *J. Catal.* **337**, 284–292 (2016).
18. Xia, S. et al. Hydrogenolysis of glycerol over $Cu_{0.4}/Zn_{5.6\text{-}x}Mg_xAl_2O_{8.6}$ catalysts: the role of basicity and hydrogen spillover. *J. Catal.* **296**, 1–11 (2012).
19. Miyazawa, T., Koso, S., Kunimori, K. & Tomishige, K. Glycerol hydrogenolysis to 1,2-propanediol catalyzed by a heat-resistant ion-exchange resin combined with Ru/C. *Appl. Catal. A: Gen.* **329**, 30−35 (2007).
20. Shinmi, Y., Koso, S., Kubota, T., Nakagawa, Y. & Tomishige, K. Modification of Rh/$SiO_2$ catalyst for the hydrogenolysis of glycerol in water. *Appl. Catal. B: Environ.* **94**, 318−326 (2010).
21. Liu, L., Kawakami, S., Nakagawa, Y., Tamura, M. & Tomishige, K. Highly active iridium–rhenium catalyst condensed on silica support for hydrogenolysis of glycerol to 1,3-propanediol. *Appl. Catal. B: Environ.* **256**, 117775 (2019).
22. Falcone, D. D. et al. Evidence for the bifunctional nature of Pt–Re catalysts for selective glycerol hydrogenolysis. *ACS Catal.* **5**, 5679–5695 (2015).
23. Fan, Y. et al. Nanoparticulate Pt on mesoporous SBA-15 doped with extremely low amount of W as a highly selective catalyst for glycerol hydrogenolysis to 1,3-propanediol. *Green Chem.* **19**, 2174−2183 (2017).
24. Huang, Z. et al. Highly dispersed silica-supported copper nanoparticles prepared by precipitation-gel method: a simple but efficient and stable catalyst for glycerol hydrogenolysis. *Chem. Mater.* **20**, 5090−5099 (2008).
25. Mallesham, B., Sudarsanam, P., Reddy, B. V. S. & Reddy, B. M. Development of cerium promoted copper–magnesium catalysts for biomass valorization: selective hydrogenolysis of bioglycerol. *Appl. Catal. B: Environ.* **181**, 47−57 (2016).
